# A secreted catalase contributes to *Puccinia striiformis* resistance to host-derived oxidative stress

**DOI:** 10.1007/s44154-021-00021-2

**Published:** 2021-12-29

**Authors:** Pu Yuan, Wenhao Qian, Lihua Jiang, Conghui Jia, Xiaoxuan Ma, Zhensheng Kang, Jie Liu

**Affiliations:** 1 State Key Laboratory of Crop Stress Biology for Arid Areas and College of Plant Protection, Northwest A&F University, Yangling, People’s Republic of China; 2 State Key Laboratory of Crop Stress Biology for Arid Areas and College of Life Sciences, Northwest A&F University, Yangling, People’s Republic of China

**Keywords:** Wheat stripe rust, *Puccinia striiformis* f. sp. *tritici*, Catalase, Reactive oxygen species, Host-induced gene silencing

## Abstract

Plants can produce reactive oxygen species (ROS) to counteract pathogen invasion, and pathogens have also evolved corresponding ROS scavenging strategies to promote infection and pathogenicity. Catalases (CATs) have been found to play pivotal roles in detoxifying H_2_O_2_ formed by superoxide anion catalyzed by superoxide dismutases (SODs). However, few studies have addressed H_2_O_2_ removing during rust fungi infection of wheat. In this study, we cloned a CAT gene *PsCAT1* from *Puccinia striiformis* f. sp. *tritici* (*Pst*), which encodes a monofunctional heme-containing catalase. PsCAT1 exhibited a high degree of tolerance to pH and temperature, and forms high homopolymers.

Heterologous complementation assays in *Saccharomyces cerevisiae* reveal that the signal peptide of PsCAT1 is functional. Overexpression of *PsCAT1* enhanced *S. cerevisiae* resistance to H_2_O_2_. Transient expression of *PsCAT1* in *Nicotiana benthamiana* suppressed Bax-induced cell death. Knockdown of *PsCAT1* using a host-induced gene silencing (HIGS) system led to the reduced virulence of *Pst*, which was correlated to H_2_O_2_ accumulation in HIGS plants. These results indicate that PsCAT1 acts as an important pathogenicity factor that facilitates *Pst* infection by scavenging host-derived H_2_O_2_.

**Supplementary Information:**

The online version contains supplementary material available at 10.1007/s44154-021-00021-2.

## Introduction

The burst of reactive oxygen species (ROS) is the early immune responses of the host to pathogens. ROS (e.g. O_2_^•^^−^, H_2_O_2_, ^•^OH, ^1^O_2_) are considered to the unavoidable toxic byproducts of aerobic metabolism and can directly damage pathogens, thus, it is necessary for pathogens to effectively scavenge host-derived ROS to establish parasitic relationships (Mittler, 2017; Guo et al., 2019; Zheng et al., 2020). ROS are removed or detoxified by an array of antioxidative enzymes such as superoxide dismutase (SOD), ascorbate peroxidase (APX), catalase (CAT), glutathione peroxidase (GPX), and peroxiredoxin (PRX) and antioxidants such as ascorbate (vitamin C), glutathione (GSH) and tocopherol (vitamin E) (Mittler et al., 2004; Foyer and Noctor, 2005).

Catalase (EC 1.11.1.6) is a type of terminal oxidase that widely exists in animals, plants and microorganisms. According to its physical and biochemical characteristics, CATs are divided into four types: typical catalase (monofunctional), catalase-peroxidase (bifunctional), non-heme catalase (pseudo-catalases) and minor catalase (Chelikani et al., 2004; Sooch et al., 2014). Monofunctional heme catalase is commonly a tetramer formed by four identical dumbbell-shaped subunits, with a heme prosthetic group at the catalytic center (Zámocký and Koller, 1999; Lee et al., 2007). Bifunctional catalase is a heme enzyme and has the catalytic activity of peroxidase (Zámocký and Koller, 1999; Kapetanaki et al., 2007). Pseudo-catalase is a heme-free catalase, which replaces the active center with a divalent manganese ion (Sooch et al., 2014). Minor catalase containing heme exhibits a very low level of catalytic activity, such as chloroperoxidase, bromoperoxidase, and catalase-phenol oxidase (Nicholls et al., 2001; Vetrano et al., 2005).

Previous studies have found that CATs are associated with cell development and differentiation, the production of metabolites (Yuan et al., 2021), and the response to oxidative stress, as an indispensable scavenger (Montibus et al., 2015). For example, in *Saccharomyces cerevisiae*, *cta1* can confer the elimination of H_2_O_2_ produced by β-oxidation, and *ctt1* is involved in the responses to oxidative and osmotic stress (Jamieson, 1998). The *catA* and *catB* from *Aspergillus nidulans* both responded to different stress conditions (such as H_2_O_2_, heat-shock and paraquat) at the stage of spores or growing hyphae, respectively (Navarro et al., 1996; Kawasaki et al., 1997). The two monofunctional CATs of *Neurospora crassa*, *NcCat1* (mainly expressed in conidia) and *NcCat3* (expressed in growing hyphae) can reduce toxic effects of oxidative stress (Michán et al., 2002; Yamashita et al., 2007). The mutant *ΔcatP* of *Beauveria bassiana* is the most sensitive to H_2_O_2_ compared to the other CAT mutants, indicating that *catP* is essential for countering defense against oxidative stress (Wang et al., 2013).

In addition, CATs play an important role during infection of fungi by effectively detoxifying hydrogen peroxide released by host. For example, *Aspergillus fumigatus* contains two mycelia-specific catalase (*CAT1*, *CAT2*) and one spore-specific catalase (*CATA*), and mycelia of the double *Δcat1 Δcat2* mutant showed reduced virulence (Calera et al., 1997; Paris et al., 2003). *CAT1*-deficient homozygous null mutant strain of *Candida albicans* was far less virulent to mice (Wysong et al., 1998). An extracellular catalase CATB in *Blumeria graminis* f. sp. *hordei* (*Bgh*) can scavenge host-derived H_2_O_2_ during infection of barley, and may contribute to pathogenicity in *Bgh* (Zhang et al., 2004). In *Magnaporthe grisea*, the catalase gene *CATB* was 600-fold up-regulated in response to exogenous H_2_O_2_ in vivo, and the *catB* mutant led to compromised pathogen fitness (Skamnioti et al., 2007). The virulence to *Spodoptera litura* larvae reduced by 33–47% in knockout mutants *ΔcatA*, *ΔcatP* and *ΔcatD* of *B. bassiana*, respectively (Wang et al., 2013). The *cat1* overexpression strain in *Metarhizium anisopliae* reduced the germination time and increased the pathogenicity to *Plutella xylostella* larvae (Morales Hernandez et al., 2010). The catalase mutant *∆KatG2* from *Fusarium graminearum*, exclusively located on the cell wall of invading hyphal cells, reduced the virulence in wheat spike infection (Guo et al., 2019). In addition, knocking out the transcription factors regulating the catalases, such as *ΔstuA* mutant in *A. snidulans* (Scherer et al., 2002) and *Δcptf1* mutant in *Claviceps purpurea* (Nathues et al., 2004), has a significant impact on virulence.

Wheat stripe rust caused by *Puccinia striiformis* f. sp. *tritici* (*Pst*) is one of the most destructive wheat diseases, resulting in serious wheat yield losses. *Pst*, an obligate biotrophic pathogen, hijacks the nutrients from host cells through haustoria, accompanied by ROS accumulation and host immunity induction (Chang et al., 2017; Wang et al., 2007). Removing host-derived ROS is crucial for *Pst* colonization in host-pathogen interactions. Previous studies have reported that two *Pst* SOD-encoding genes, *PsSOD1* (a potential Zn-only SOD) and *PsSOD2* (a Cu-only SOD), were deployed for counter defense against host-derived oxidative stress (Liu et al., 2016; Zheng et al., 2020). SODs catalyze the conversion of superoxide anions into molecular oxygen and hydrogen peroxide, and then CATs convert hydrogen peroxide into water and molecular oxygen, constituting the vital line of cellular defenses against ROS damages (Guo et al., 2019). However, few studies are available regarding the role of CAT during *Pst* infection of wheat. In this study, an extracellular CAT gene from *Pst*, *PsCAT1*, exhibiting a high expression level during the early stage of *Pst* infection, was characterized. Secretion and biochemical characteristics of PsCAT1 were determined by heterologous expression. In addition, the function of PsCAT1 was identified through overexpression and a host-induced gene silencing (HIGS) system. Our results indicate that PsCAT1 served as a virulence factor to promote *Pst* infection of wheat by counteracting host-derived oxidative stress.

## Results

### Cloning and expression analysis of *PsCAT1*

Extracellular antioxidant enzymes play pivotal roles during infection of pathogens (Liu et al., 2016). In the *Pst* genome, four genes annotated as CATs were designated *PsCAT1*, *PsCAT2, PsCAT3, PsCAT4.* PsCAT1, PsCAT2 and PsCAT3 were found to contain the signal peptides using signalP 4.1 (Fig. S1). In addition, semi-quantitative reverse transcription PCR (sqRT-PCR) analysis showed that *PsCAT1* was continuously expressed at a high level in urediniospores and infection structures (Fig. S2). *PsCAT2* showed a lower transcript level during infection compared with ungerminated urediniospores (Fig. S2), while *PsCAT3* was not expressed in all developmental early stages (Fig. S2). Thus, *PsCAT1* was subjected to more detailed functional characterization as described in the following sections.

*PsCAT1* was amplified by RT-PCR using a CYR31-infected Suwon 11 (Su11) cDNA sample as a template. The open reading frame (ORF) of *PsCAT1* consists of 1557 nucleotides and is predicted to encode a polypeptide of 518 amino acids with a calculated molecular weight of 62,280 Da, an isoelectric point (pI) of 8.63. In addition, two domains (catalase and catalase-related immune-responsive domains), and active sites of Asparagine and Histidine for metal iron binding were identified in the PsCAT1 protein sequence by HMMER analysis (Fig. S3).

The 518 amino acid sequence was used as a query sequence to search the most up-to-date databases (NCBI). Homologous proteins from other fungi with the highest similarities to PsCAT1 were determined. The protein sequence showed 63.23% identity with the CAT protein from *Puccinia graminis* f. sp. *tritici* CRL 75–36–700-3 (GenBank accession number XP_003329286.2), and 42.45% identity with the CAT protein from *Puccinia sorghi* (GenBank accession number KNZ48073.1). The phylogenetic analysis of PsCAT1 with homologous proteins from other fungi revealed that PsCAT1 displays greater similarity to CATs from basidiomycetous fungi, especially rust fungi, compared with those from ascomycetous fungi (Fig. S4). These results indicate *PsCAT1* possibly encodes a typical catalase.

### Biochemical characterization of PsCAT1

**Fig. 1:**
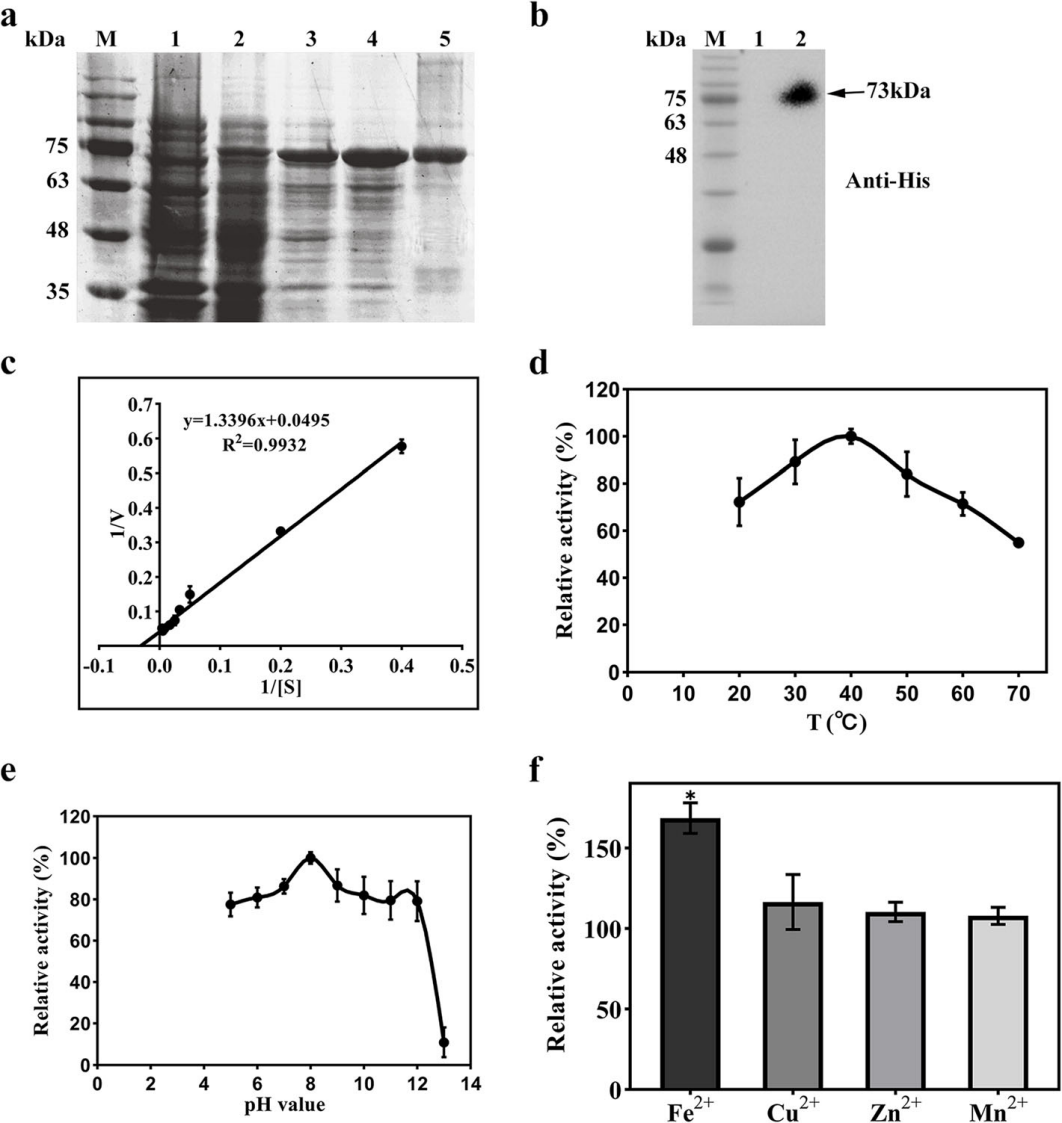
Purification and biochemical characterization of PsCAT1. **a**. The SDS-PAGE profiles of PsCAT1 in *E. coli* BL21(DE3) plysS. Lane 1, uninduced *E. coli* cell lysates harboring pET15b-SUMO-*PsCAT1*; lane 2, *E. coli* cell lysates harboring pET15b-SUMO-*PsCAT1* induced by IPTG; lane 3 and 4, soluble and insoluble fractions from the cell culture expressing *PsCAT1*; lane 5, the purified PsCAT1 fusion proteins; M, marker. **b**. Western blotting analysis of the purified PsCAT1 fusion proteins. Lane 1, uninduced *E. coli* cell lysates harboring pET15b-SUMO-*PsCAT1*; lane 2, the purified PsCAT1 fusion proteins; M, marker. **c** Lineweaver-Burk plot for PsCAT1. **d** and **e**. Thermal and pH stability of the purified PsCAT1 proteins. **f**. Effects of metal ions on the activity of the purified PsCAT1 proteins. Fe^2+^, Fe_2_(SO_4_)_3_; Cu^2+^, CuSO4•5H_2_O; Zn^2+^, ZnSO_4_•7H_2_O; Mn^2+^, MnSO_4_•H_2_O. Asterisks indicate a significant difference (*P* < 0.05) compared with the control using Student’s *t*-test

The recombinant plasmid pET15b-SUMO-*PsCAT1* was transformed to *E. coli* BL21(DE3) plysS to induce the expression of the recombinant PsCAT1 protein using 0.4 mM IPTG, as shown in the SDS-PAGE profiles (Fig. 1a). Enzymatic characterization of PsCAT1 were performed with the purified PsCAT1 protein obtained by immobilized-nickel affinity chromatography. SDS-PAGE and Western blotting analysis (anti-His antibody) showed that the expressed fusion protein was about 73 kDa containing the His 6 in series with the SUMO tag (~ 12 kDa) (Fig. 1a and Fig. 1b), which is consistent with the predicted molecular weight of PsCAT1.

The Michaelis-Menten kinetics of PsCAT1 was then measured using the Lineweaver-Burk plot method (Chang et al., 2017). *K*m and *V*max were determined to be 27.06 mM and 20.20 mM mg^− 1^ min^− 1^ under optimal conditions (Fig. 1c). The optimum temperature was approximately 40°C, and a high enzyme activity was measured at 70°C, indicating that PsCAT1 has a strong tolerance to high temperature (Fig. 1d). The pH optimum was determined to be approximately pH 8.0. Only slight change was observed in enzyme activity, from pH 5 to 12, whereas the enzyme was almost inactivated at pH 13 (Fig. 1e). In addition, metal cations also showed different effects on the enzyme activity of PsCAT1. Including 0.2 mM Fe^2+^ in the reaction can increase the enzyme activity by 68%, whereas Zn^2+^, Cu^2+^ and Mn^2+^ have no obvious effect on the PsCAT1 enzyme activity (Fig. 1f).

### PsCAT1 potentially forms homopolymers

**Fig. 2:**
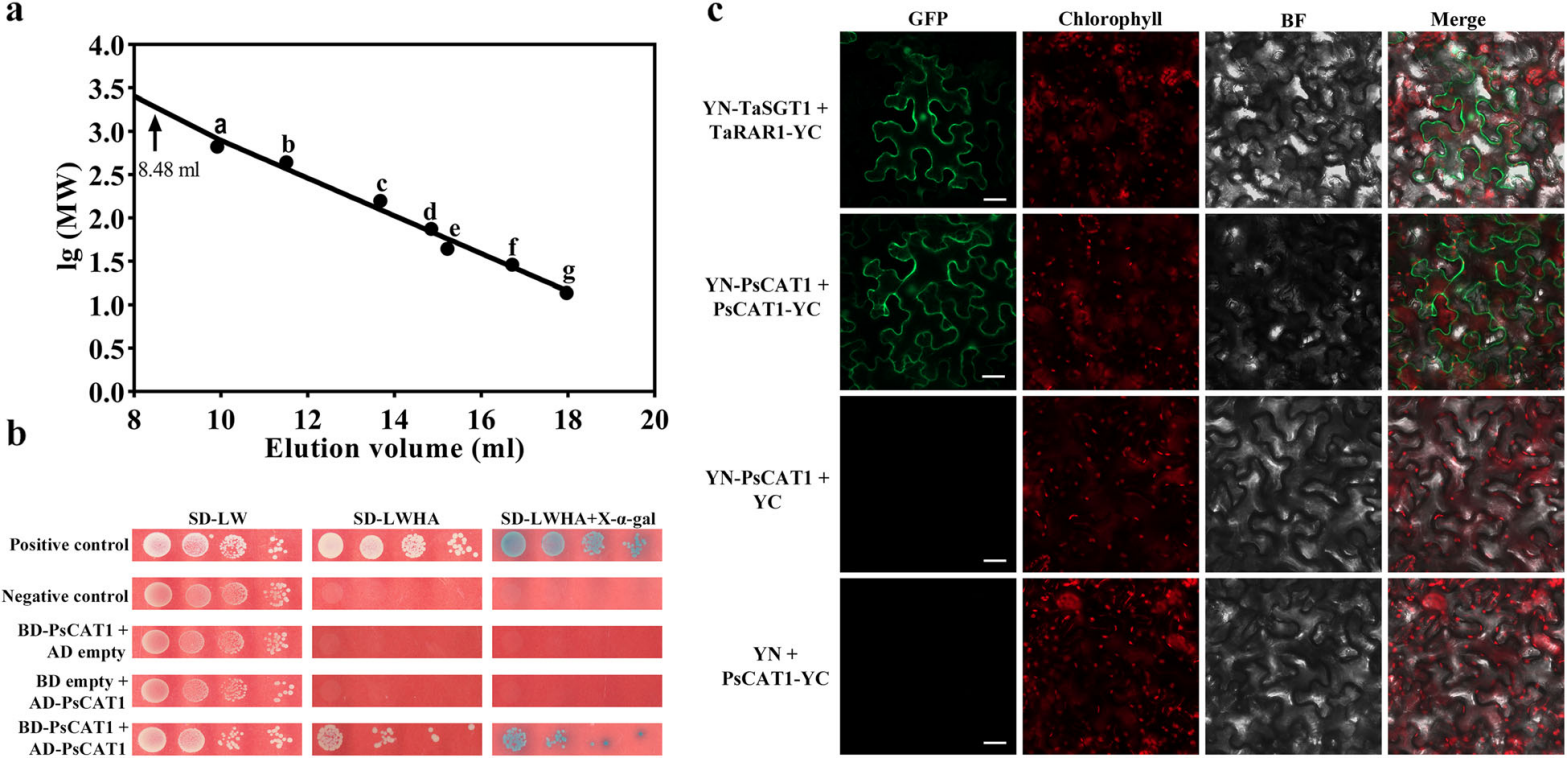
Determination of the molecular weight of PsCAT1. **a**. Gel filtration was performed on a Superdex-200 column. Standard proteins: a Thyroglobulin (670 kDa, 9.92 ml), **b** Ferritin (440 kDa, 11.51 ml), **c** Globulin (158 kDa, 13.68 ml), **d** Conalbumin (75 kDa, 14.85 ml), **e** Ovalbumin (44 kDa, 15.22 ml), **f** Carbonic anhydrase (29 kDa, 16.72 ml), **g** Ribonuclease A (13.7 kDa, 17.97 ml). The arrow indicates the elution volume of PsCAT1. **b**. Yeast two-hybrid assay for homo-oligomerization of PsCAT1. For yeast transformants, four serial 1:10 dilutions are shown for each combination. The *S. cerevisiae* harboring pGBKT7-Lam and pGADT7-T, pGADT7 and pGBKT7-*PsCAT1*, and pGBKT7 and pGADT7-*PsCAT1* were used as the negative controls. Co-transformation with pGBKT7–53 and pGADT7-T acted as a positive control. L, Leu; W, Trp; H, His; A, Ade. **c**. In vivo BiFC analysis of PsCAT1 homo-oligomerization. Yn-TaSGT1 + TaRAR1-Yc is the positive control; Yn-PsCAT1 + PsCAT1-Yc is shown in the middle panel; Yn-PsCAT1 + Yc and Yn + Yc-PsCAT1 acted as the negative controls. Agrobacterium-mediated transient expression of indicated constructs in *N. benthamiana* leaves. Bright-field (BF), GFP fluorescence and Chlorophyll fluorescence images were taken by microscopy and merged. Scale bars = 10 μm

To investigate the polymerization of PsCAT1, the purified PsCAT1 protein sample was subjected to size exclusion chromatography. The native molecular weight of PsCAT1 was determined to be 1690.27 kDa (Fig. S5a; Fig. 2a), which was approximately 23 times as high as that of the PsCAT1 monomer. The sample in absorption peak was verified as the target protein by Western blotting (Fig. S5b). As shown in Fig. 2a, the elution peak of 8.48 ml is beyond the upper limit of the calibration curve, the molecular weight of the polymer cannot be accurately measured. Then, the purified PsCAT1 protein denatured with urea was separated by gel filtration chromatography, again. The result showed that there were two absorption peaks, 8.35 ml (a high polymer) and 14.69 ml (a monomer), respectively (Fig. S5c). Western blotting analysis further indicated that the two absorption peaks are the different forms of the target protein (Fig. S5d). These results suggest that PsCAT1 is a high polymer.

To further identify the self-interaction of PsCAT1, the recombinant plasmids pGADT7-*PsCAT1* and pGBKT7-*PsCAT1* were co-transformed into *S. cerevisiae* strain AH109. Co-transformations with pGBKT7-Lam and pGADT7-T, pGADT7 and pGBKT7-*PsCAT1*, and pGBKT7 and pGADT7-*PsCAT1* were used as the negative controls, whereas the transformants containing pGBKT7–53 and pGADT7-T acted as a positive control. The interactions were assessed by the survival of yeast on the selection medium SD/−Leu-Trp-His-Ade (SD-LWHA) and the production of β-galactosidase. As shown in Fig. 2b, reporter activation suggested that PsCAT1 is capable of interacting with itself.

In addition, bimolecular fluorescence complementation (BiFC) assay in transiently transformed *N. benthamiana* leaves was performed to confirm the polymerization of PsCAT1. Similar to the positive control (Fig. 2c), strong fluorescence signals were observed when agrobacteria carrying pSPYNE(R)173-*PsCAT1* and pSPYCE(M)-*PsCAT1* were co-infiltrated into *N. benthamiana* leaves (Fig. 2c). However, with the co-expression of pSPYNE(R)173-*PsCAT1* and the empty pSPYCE(M) vector, or pSPYCE(M)-*PsCAT1* and the empty pSPYNE(R)173 in *N. benthamiana* leaves, no fluorescence was visualized (Fig. 2c). These results indicate that PsCAT1 can form homopolymers.

### Functional validation of the signal peptide of PsCAT1

**Fig. 3:**
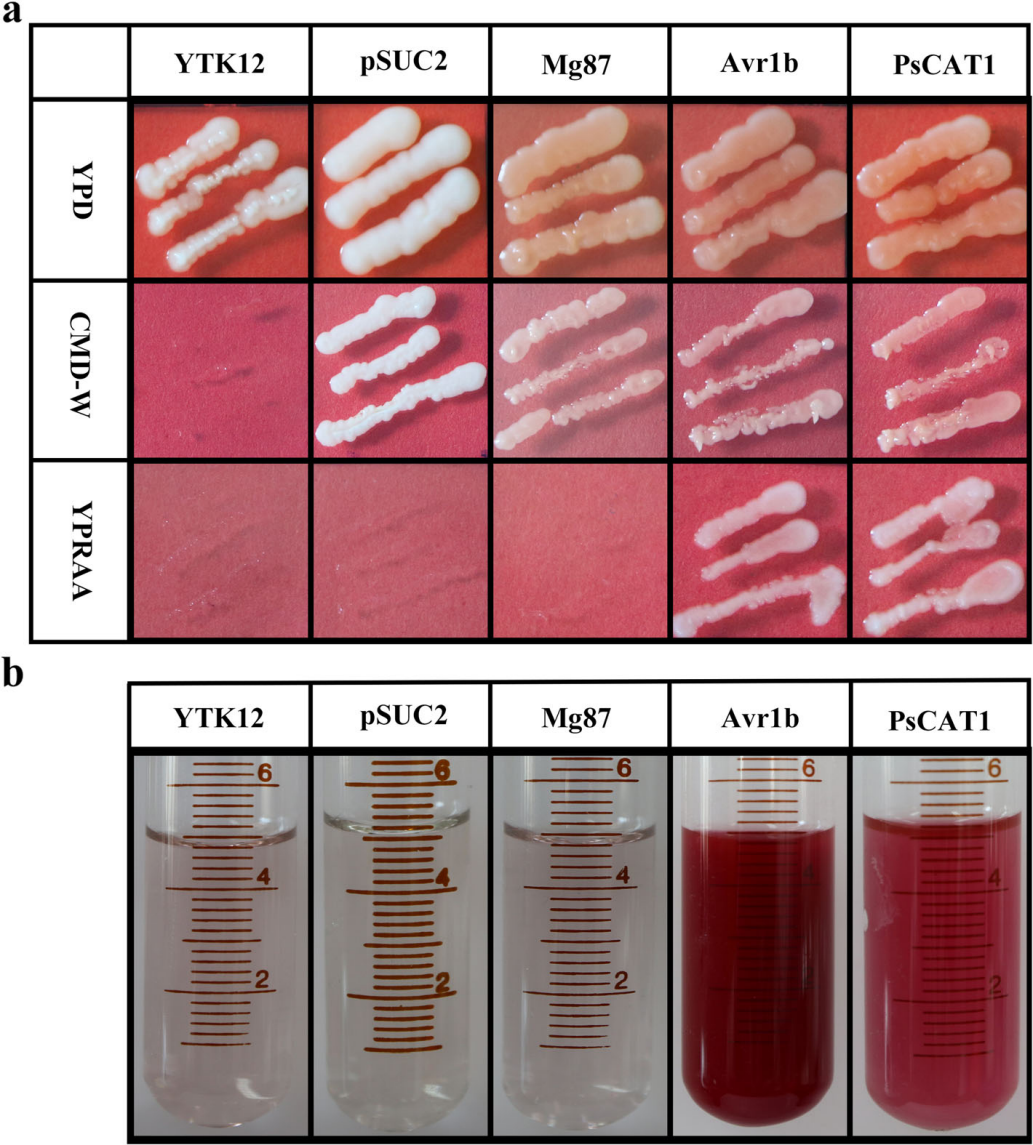
Functional validation of the signal peptide of PsCAT1. **a**. The PsCAT1 or Avr1b signal peptides or the first 25 amino acids of Mg87 were fused in-frame to the invertase sequence in the pSUC2 vector and transformed into the yeast YTK12 strain. Controls include the untransformed YTK12 strain and YTK12 carrying the pSUC2 vector. Strains that are unable to secrete invertase can grow on CMD-W media but not on YPRAA media **b**. The color reaction was used to verify the function of the PsCAT1 signal peptide. The extracellular invertase enzyme activity was detected by the reduction of 2,3,5-Triphenyltetrazolium Chloride (TTC) to insoluble red colored 1,3,5-Triphenylformazan (TPF)

To functionally validate the SignalP 4.1 predictions, the signal peptide of PsCAT1 was tested using the *S. cerevisiae*. Firstly, the recombinant plasmids pSUC2-*PsCAT1*_*sp*_, pSUC2-*Avr1b*_*sp*_, pSUC2-*Mg87*_*1–75*_ and the empty vector pSUC2 were transformed into the invertase mutated yeast strain YTK12, respectively. pSUC2-*Mg87*_*1–75*_ and the empty pSUC2 vector were used as the negative controls. pSUC2-*Avr1b*_*sp*_ served as a positive control. The results showed that both the pSUC2-*PsCAT1*_*sp*_ and pSUC2-*Avr1b*_*sp*_ fused constructs enabled YTK12 to grow on CMD-W media (yeast can grow without invertase secretion) and YPRAA media (yeast can grow only when invertase is secreted) (Fig. 3a). Additionally, in the color reaction, the yeast strains transformed by pSUC2-*PsCAT1*_*sp*_ and pSUC2-*Avr1b*_*sp*_ restored the secretion function of invertase, thus the invertase enzymatic activity can be detected by the reduction of 2,3,5-Triphenyltetrazolium chloride (TTC) to insoluble red colored 1,3,5-Triphenylformazan (TPF) (Fig. 3b). These results confirmed that the signal peptide of PsCAT1 was functional.

### PsCAT1 enhances *S. cerevisiae* resistance to ROS

**Fig. 4:**
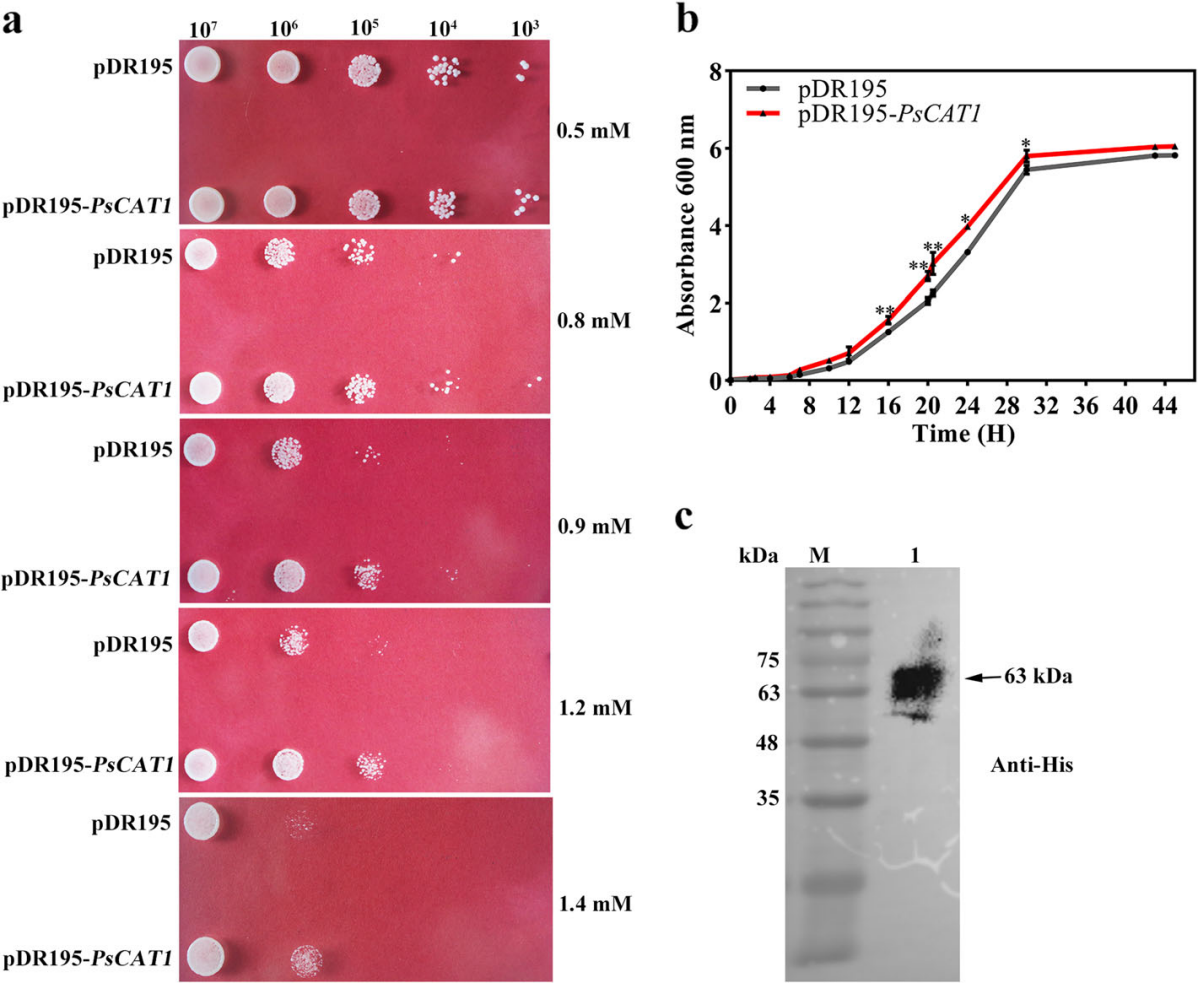
Overexpression of *PsCAT1* in *S. cerevisiae*. **a**. A spot assay of the *S. cerevisiae* strain YNL241C harboring pDR195 or pDR195-*PsCAT1* on SD/−Ura plates with H_2_O_2_. **b**. Liquid culture assay of the *S. cerevisiae* strain carrying pDR195-*PsCAT1* in SD/−Ura media with 1.2 mM H_2_O_2_. The *S. cerevisiae* harboring pDR195 empty vector was used as the control. Overexpression of *PsCAT1* significantly enhanced *S. cerevisiae* resistance to ROS stress compared with the control. **c**. Western blotting analysis of *PsCAT1* expression in *S. cerevisiae*

To identify the function of *Ps*CAT1, the empty vector pDR195 and the recombinant plasmid pDR195-*PsCAT1* were transformed into a *S. cerevisiae* strain YNL241C, respectively. The growth of the positive transformants was monitored on synthetic complete (SC) media containing different concentrations of H_2_O_2_. The result showed that PsCAT1 conferred enhanced resistance of *S. cerevisiae* to H_2_O_2_ compared with the control (Fig. 4a). Additionally, the growth curves of the above-mentioned two strains were constructed in liquid SC media with 1 mM H_2_O_2_. As shown in Fig. 4b, the *S. cerevisiae* strain carrying pDR195-*PsCAT1* grew significantly faster than the control harboring the empty pDR195 vector. To further confirm that PsCAT1 was secreted extracellularly to remove exogenous H_2_O_2_, the culture supernatants of the *S. cerevisiae* with pDR195-*PsCAT1* were determined using Western blotting. A ~ 63 kDa-band was clearly exhibited (Fig. 4c), which is in accordance with the above result that PsCAT1 possess functional signal peptide.

### PsCAT1 suppresses Bax-induced cell death by scavenging ROS

**Fig. 5:**
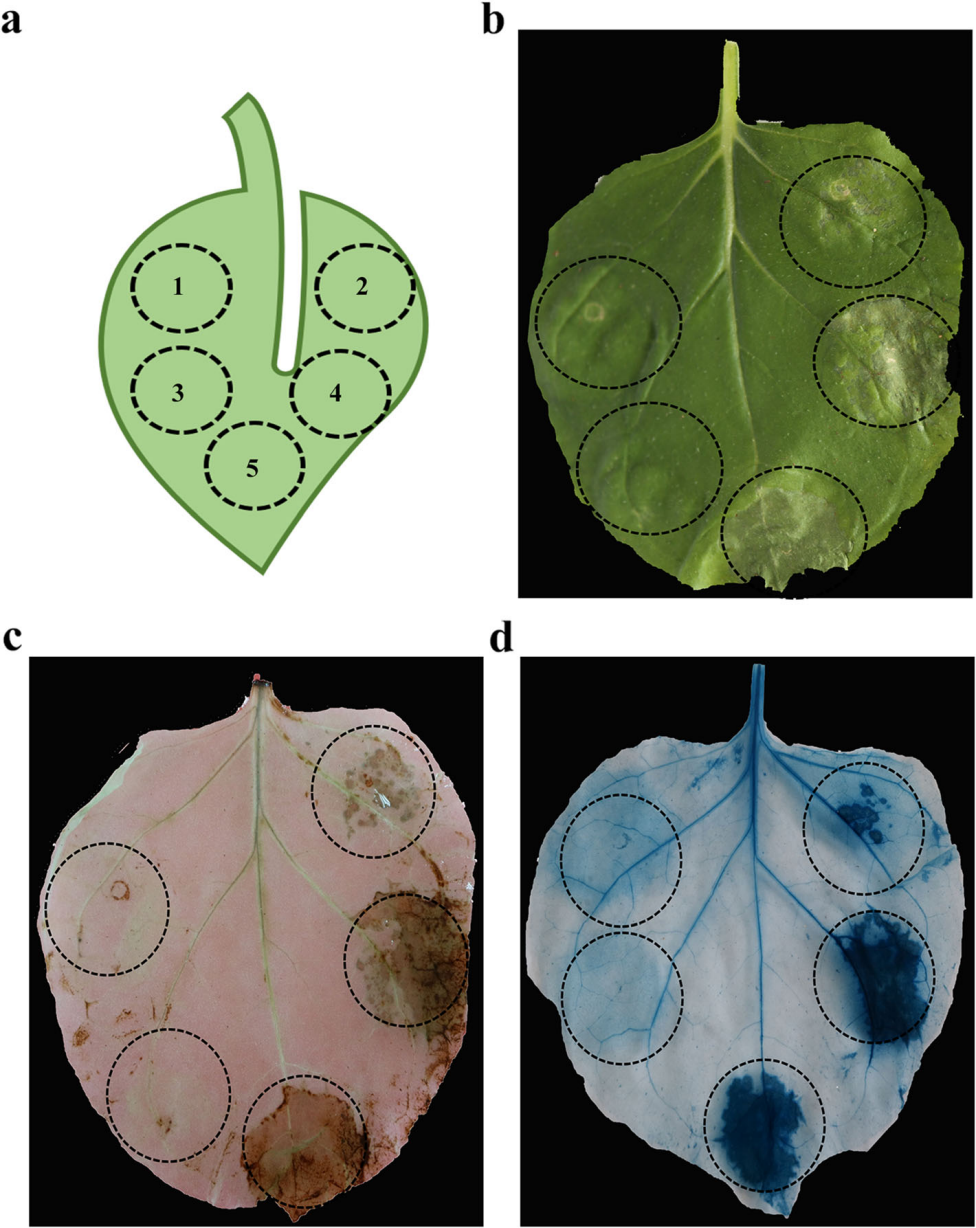
Transient expression of *PsCAT1* in *N. benthamiana*. **a**. Five injection sites on tobacco leaves. 1, PsCAT1; 2, PsCAT1 + Bax (infiltrated 24 h later); 3, empty vector; 4, empty vector + Bax (infiltrated 24 h later); 5, Bax. **b**. Tobacco leaves were infiltrated with *A. tumefaciens* cells carrying *PsCAT1*, an empty vector or Bax alone (circles 1, 3, 5), infiltrated with *A. tumefaciens* cells containing *PsCAT1* or empty vector and followed 24 h later by a second infiltration of *A. tumefaciens* cells carrying Bax (circles 2, 4). Photos were taken from 4 days after the second infiltration. **c**. H_2_O_2_ accumulation in *N. benthamiana* leaves was determined by DAB staining. **d**. Cell necrosis was determined by Trypan Blue staining

To clarify whether PsCAT1 functions in suppressing the host defence responses, *PsCAT1* was transiently expressed in tobacco leaves. When *N. benthamiana* leaves were infiltrated with *A. tumefaciens* strains individually carrying PVX-*PsCAT1* or PVX-*GFP* (the negative control, NC), no cell death was observed (Fig. 5b; circle 1, 3); tobacco leaves infiltrated with Bax (a pro-apoptotic protein from mouse that triggers a hypersensitive response (HR)-like cell death response in plants) + NC (Fig. 5b; circle 4) or Bax only (Fig. 5b; circle 5) both showed a similar cell death phenotype (after 4 days). However, when PsCAT1 was infiltrated prior to Bax for 24 h, cell death was significantly suppressed (Fig. 5b; circle 2). Accordingly, H_2_O_2_ production in *N. benthamiana* leaves was detected by DAB staining. The result showed that the expression of *PsCAT1* led to less H_2_O_2_ generated in the injection site circle 2 compared with circles 4 and 5 (Fig. 5c). In addition, trypan blue staining was used to assess cell death. As a result, the injection site circles 4 and 5 have a large number of necrotic cells, in contrast, only mild necrosis was observed in circle 2 (Fig. 5d). To confirm that PsCAT1, GFP and Bax were successfully expressed in *N. benthamiana* leaves, Western blotting analysis was performed. As shown in Fig. S6, anti-HA antibody, anti-GFP antibody and anti-Bax antibody can detect the expression of the HA-PsCAT1, GFP and Bax proteins, respectively. These results indicate that PsCAT1 can function as a ROS scavenger to counteract the host defence responses.

### Silencing of *PsCAT1* by HIGS reduces the virulence of *Pst* infection of wheat

**Fig. 6:**
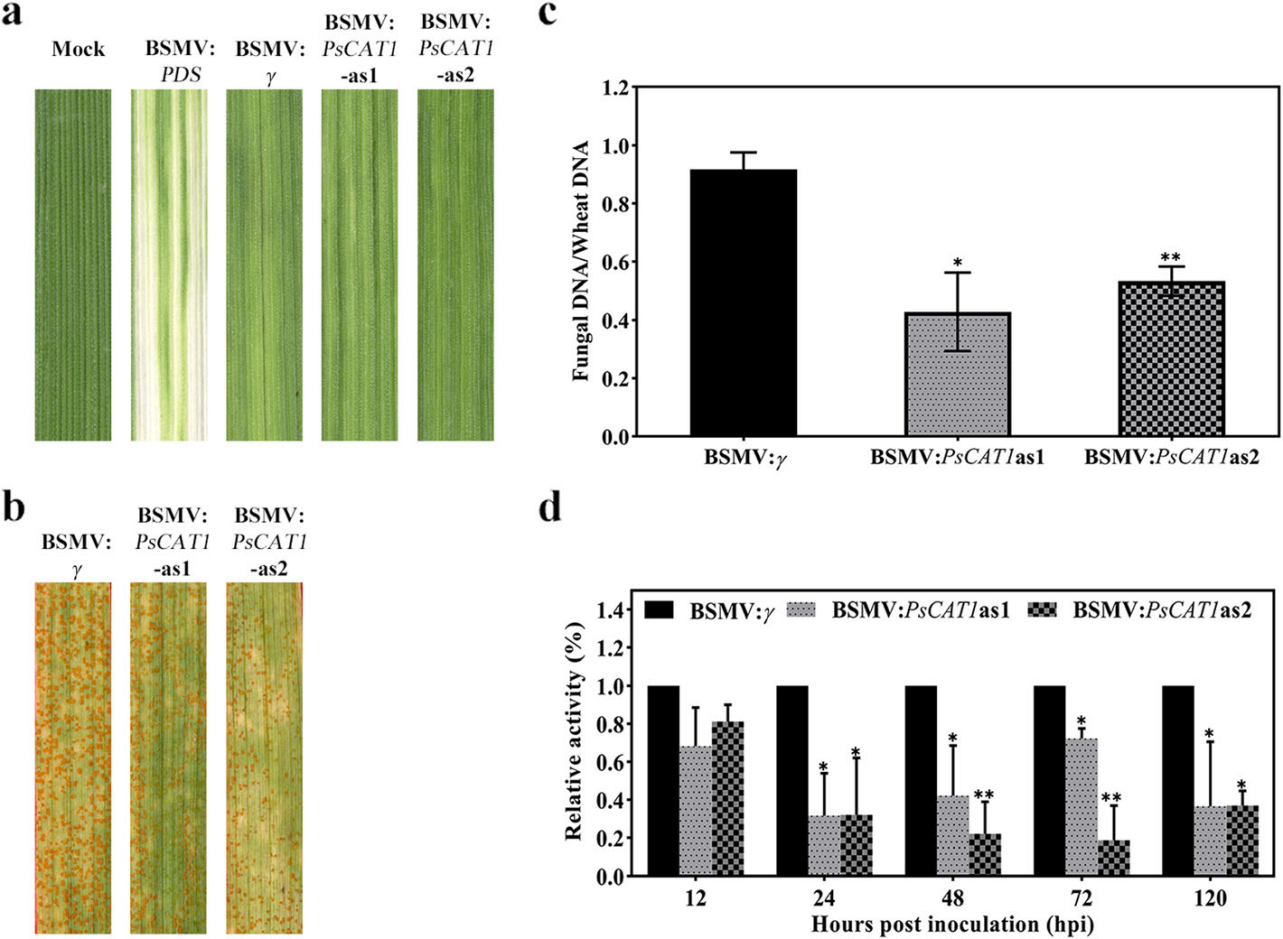
Silencing of *PsCAT1* in the wheat-*Pst* interaction using HIGS leads to reduced virulence. **a**. Mild chlorotic mosaic symptoms were observed on the fourth leaves of seedlings at 10 dpi with BSMV, and bleaching was evident on the fourth leaves of plants infected by BSMV:*TaPDS*. Mock, wheat leaves inoculated with FES buffer. **b**. Disease phenotypes of the fourth leaves pre-inoculated with BSMV and then challenged with CYR31. **c**. Fungal biomass measurements using real-time PCR analysis of total DNA extracted from the wheat leaves infected by CYR31 at 15 dpi. Ratio of total fungal DNA to total wheat DNA was assessed using the wheat gene *TaEF-1α* and the *Pst* gene *PstEF1*. **d**. Silencing efficiency assessment of *PsCAT1* in *Pst*. Wheat leaves inoculated with BSMV:*γ* and sampled after inoculation with CYR31 were used as the controls. The data were normalized to the expression level of *TaEF-1α*. The mean ± SD from four independent samples is presented. Asterisks indicate a significant difference (*P* < 0.05) using Student’s *t*-test

To investigate the function of *PsCAT1* during *Pst* infection of wheat, we used the HIGS technique to silence *PsCAT1* in *Pst*. Wheat seeding inoculated with barley stripe mosaic virus (BSMV) showed mild chlorotic mosaic symptoms at 10 days after inoculation (dai), and a photo-bleaching phenotype also observed in the *TaPDS* (*TaPDS*: wheat phytoene desaturase gene)-silenced wheat leaves (Fig. 6a). After *Pst* inoculation, the wheat plants inoculated with BSMV-*PsCAT1*as1 (carrying a 142-bp fragment of *PsCAT1*) and BSMV-*PsCAT1*as2 (carrying a 133-bp fragment of *PsCAT1*) exhibited a significant reduction in sporulation compared with the control BSMV-*γ*-infected wheat leaves at 15 dai (Fig. 6b).

Subsequently, fungal biomass in the host tissue was measured as described by Huai et al. (2019), to examine whether the reduction in sporulation was related to hyphal growth restriction. In *Pst*-infected wheat leaves inoculated with BSMV-*PsCAT1*as1 and BSMV-*PsCAT1*as2, the fungal biomass was obviously reduced by 48% and 38% respectively, compared with the controls inoculated with BSMV-*γ* (empty BSMV) (Fig. 6c). This result demonstrated that fungal development was impeded, probably as a result of the the silencing of *PsCAT1.*

To clarify whether *PsCAT1* was successfully silenced, the relative transcript level of *PsCAT1* was measured using qRT-PCR in *Pst*-infected wheat leaves. The results showed that the *PsCAT1* transcript in BSMV-*PsCAT1*as1-inoculated leaves was reduced by 68%, 57%, 28% and 63% at 24, 48, 72 and 120 hpi respectively; in leaves inoculated with BSMV-*PsCAT1*as2, the *PsCAT1* expression level was decreased by 68%, 77%, 81% and 63% respectively, compared with BSMV-*γ*-infected wheat leaves (Fig. 6d). These results indicate that the expression of *PsCAT1* was efficiently knocked down by BSMV-HIGS.

### HIGS of *PsCAT1* impaired fungal growth and enhanced H_2_O_2_ accumulation

**Fig. 7:**
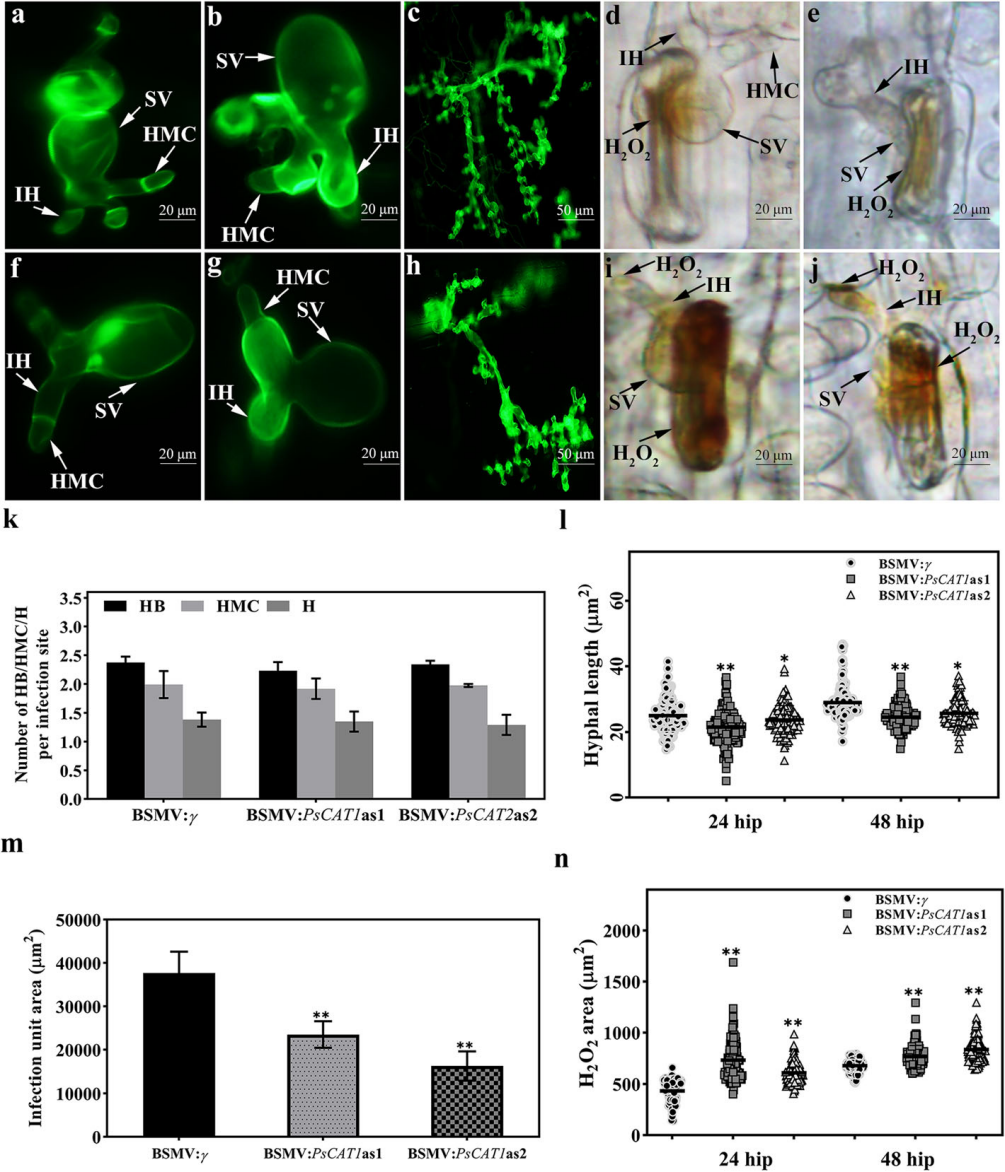
Histological observation of fungal growth and host response in BSMV:*γ* and recombinant BSMV inoculated wheat leaves infected with CYR31. **a-e**. Fungal growth at 24 hpi (**a**) or 48 hpi (**b**), infection unit area at 120 hpi (**c**), H_2_O_2_ accumulation at 24 hpi (**d**) or 48 hpi (**e**) in BSMV:*γ* infected plants. **f-j**. Fungal growth at 24 hpi (**f**) or 48 hpi (**g**), infection unit area at 120 hpi (**h**), H_2_O_2_ accumulation at 24 hpi (**i**) or 48 hpi (**j**) in BSMV:*PsCAT1*-infected plants, H_2_O_2_ accumulation was determined using DAB staining. **k**. The average number of HB, HMC and H showed no significant difference in HIGS plants infected by CYR31 compared with the control at 24 hpi. **l**. Hyphal length, which is the average distance from the junction of the substomatal vesicle and the hypha to the tip of the hypha, was clearly decreased in HIGS plants infected by CYR31 at 24 hpi. **m**. The infection unit area at 120 hpi per infection unit was significantly reduced in HIGS plants infected by CYR31. **n**. A significant increase in ROS accumulation was observed in CYR31-infected HIGS plants at 24 hpi. Values represent the means ± SD of three independent samples. Differences were assessed using Student’s *t*-test. Asterisks indicate *P* < 0.05. SV, substomatal vesicle; HMC, haustorial mother cell; IH, infection hypha; HB, hyphal branch; H, haustoria

To determine PsCAT1 contribution to *Pst* pathogenicity, histological changes in the pathogen were observed in HIGS wheat plants infected with *Pst*, based on staining with wheat germ agglutinin (WGA). As shown in Fig. 7k and Fig. S6a, the number of hyphal branches (HB), haustorial mother cells (HMC) and haustoria (H) in BSMV-*PsCAT1*as1 or BSMV-*PsCAT1*as2 inoculated wheat plants infected with *Pst* were similar (*P* > 0.05) to those of the control at 24 and 48 hpi, respectively; whereas the hyphal length has decreased significantly (Fig. 7a, b, f, g and l). Moreover, there was no obvious difference in the formation of secondary hyphae compared with the control at 24 and 48 hpi (Fig. S6b), while the infection area was significantly reduced compared to control at 120 hpi (Fig. 7c, h and m). Furthermore, DAB staining was performed to determine the effect of silencing PsCAT1 on H_2_O_2_ accumulation in response to *Pst* infection in wheat. The results showed that H_2_O_2_ accumulation was significantly increased, especially in the anterior part of the HMC, in the wheat seedings inoculated with BSMV-*PsCAT1*as1 and BSMV-*PsCAT1*as2 compared with the control plants at 24 and 48 hpi (Fig. 7d, e, i, j and n). These results revealed that the *PsCAT1*-silenced *Pst* severely affects its elimination of host-derived H_2_O_2_, resulting in blocked fungal growth.

## Discussion

Higher eukaryotes use ROS originated from the oxidative burst to eliminate invading pathogens. During the coevolution of pathogens and their hosts, pathogens have coopted the antioxidant enzymes and molecules for normal ROS removing to evade oxidative killing so that survival and persistence are ensured (Cuéllar-Cruz et al., 2008). Although CATs, as a kind of key antioxidant enzymes, play a pivotal role in fungal development and exogenous stress responses, little information is available regarding the role of CATs in plant-pathogen interactions. In this study, a *Pst* CAT-encoding gene (*PsCAT1*) was cloned for the first time. Heterologous expression of *PsCAT1* could confer enhanced resistance to H_2_O_2_, while knockdown of *PsCAT1* in the wheat-*Pst* interaction led to fungal growth restriction. These results indicate that PsCAT1 functions as a virulence factor to promote *Pst* infection.

Numerous studies have found that CATs exhibited high activity over a broad pH and temperature range. For example, the CAT from *Rhodospirillum rubrum* S1 had a high activity in pH range from 5.0 to 11.0 and temperature range from 20 to 60°C (Lee et al., 2007). A CAT, KatP, from *Pigmentiphaga* sp. DL-8 remained active and stable in a wide pH-stable range of 4.0–11.0 and the enzyme activity can be detected at 5–70°C (Dong et al., 2015). High tolerance of CATs to temperature (25 to 50°C) and pH (4.0 to 11.5) was also found in *Corynebacterium glutamicum* (Yang et al., 2020). In the present study, PsCAT1 was found to display strong tolerance to temperature and pH. Considering that PsCAT1 contains a functional signal peptide and is secreted into the host-pathogen interface to remove ROS, this kind of tolerance capability of PsCAT1 may ensure a high efficiency of eliminating the host-derived H_2_O_2_ even under intricate environmental conditions.

Monofunctional CATs normally exist as a dumbbell-shaped tetramer of four identical subunits, with a molecular weight of 200–340 kDa, and with a haem prosthetic group at the catalytic center (Sooch et al., 2014). Here, the polymerization of PsCAT1 was determined using size exclusion chromatography. The self-interaction of PsCAT1 in vivo was further confirmed by yeast two-hybrid and BiFC assays. By contrast, the molecular weight is approximately 23 times as high as that of the PsCAT1 monomer, which is inconsistent with the previously reported CATs. These results suggest that *PsCAT1* probably encodes a novel monofunctional heme catalase functioning as a high polymer. During wheat-*Pst* interactions, the formation of homopolymers appears to facilitate increasing enzymatic activity of PsCAT, resulting in faster ROS scavenging and establishment of parasitic relationships.

Extracellular CATs have been shown to endow elevated resistance to exogenous stress responses (Garre et al., 1998). For example, Cuéllar-Cruz et al. (2008) found that high resistance to oxidative stress in the fungal pathogen *Candida glabrata* is mediated by a single catalase, Cta1p. The gastric pathogen *Helicobacter pylori* catalases protect the bacterium against oxidative stress (Benoit and Maier, 2016). Heterologously expressed *Debaryomyces hansenii DhCTA1* and *DhCTT1* genes conferred enhanced tolerance of *S. cerevisiae* to oxidative stress (González et al., 2020). In the present study, *PsCAT1* was overexpressed in *S. cerevisiae* treated with exogenous ROS. The results showed that *PsCAT1* could enhance *S. cerevisiae* resistance to exogenous H_2_O_2_, indicating that PsCAT1 can be secreted from the *S. cerevisiae* cells and scavenge exogenous ROS. In addition, transient expression in tobacco significantly suppressed Bax-induced cell death. Therefore, it is a reasonable inference that highly-expressed and secreted PsCAT1 is in favor of *Pst* infection, since ROS elimination in the host-pathogen interface not only could protect *Pst* from oxidative damage, but also may suppress HR-like cell death of host cells induced by ROS.

Catalases have been found to contribute to pathogen virulence by participating in ROS removing. For example, in *Xanthomonas oryzae* pv. *oryzae* (*Xoo*). Deletion of the CAT gene *catB* drastically impaired bacterial viability in the presence of extracellular H_2_O_2_ and reduced CAT activity, demonstrating that CatB contributes to H_2_O_2_ detoxification in *Xoo*. In addition, *ΔcatB* displayed shorter bacterial blight lesions and reduced bacterial growth in rice compared to the wild-type stain, indicating that *CatB* plays essential roles in the virulence of *Xoo* (Yu et al., 2016). Aflatoxin production and virulence were significantly decreased in the *cta1* deletion mutant of *Aspergillus flavus* compared with the WT and complementary strains (Zhu et al., 2020). While the extracellular CAT KatG2 in the rice blast fungus *Magnaporthe oryzae* exhibits a moderate contribution to infection during the early stages (Tanabe et al., 2011). In this study, a BSMV-HIGS system was used to identify the role of *PsCAT1* in the wheat-*Pst* interaction. The reduced disease symptoms in HIGS wheat seedlings infected by CYR31 suggested that the knockdown of *PsCAT1* could reduce the virulence of *Pst*. In addition, H_2_O_2_ accumulation was obviously increased, and the fungal development was blocked. Previous studies have shown that ROS, especially H_2_O_2_, are highly toxic to pathogens (Mittler, 2017). Therefore, we infer that accumulation of host-derived H_2_O_2_ in HIGS plants could impede fungal development during *Pst* infection, resulting in a decrease in the number of uredia. In addition, two extracellular SODs PsSOD1 and PsSOD2 have been identified as important virulence factors by catalyzing the conversion of superoxide anions to molecular oxygen and hydrogen peroxide (Liu et al., 2016; Zheng et al., 2020), while produced H_2_O_2_ is further detoxified by PsCAT1. Thus, based on the present results, an extracellular antioxidant enzyme system from *Pst* against host-derived ROS may be determined.

In summary, the present study revealed that PsCAT1, a heme-containing catalase, served as a virulence factor and potentially secreted during wheat-*Pst* interactions to contribute to *Pst* infection by scavenging host-derived ROS.

## Materials and methods

### Experimental materials and growth conditions

Wheat (*Triticum aestivum* L.) seedlings of the cultivar Suwon 11 (Su11) and *N. benthamiana* were grown in a greenhouse under 8/16 h night/day conditions at 16°C and 22°C, respectively. Fresh urediniospores of the *Pst* pathotype CYR31 were collected from the infected wheat leaves for the wheat-*Pst* interaction study. To measure the expression levels of *PsCAT1*, *PsCAT2* and *PsCAT3* in the *Pst*-infected wheat leaves, the leaf tissues were sampled at 12 h, 24 h, 36 h and 48 h and stored at -80°C for RNA extraction.

### Cloning and sequence analysis

The sequences of all *CATs* from *Pst* were derived from the CYR32 genome (Zheng et al., 2013). Protein domains were analyzed using HMMER software (http://www.ebi.ac.uk/Tools/hmmer/). Signal peptide prediction was performed using SignalP 4.1 Server software (http://www.cbs.dtu.dk/services/SignalP/). The physicochemical properties of PsCAT1 were determined by the Compute pI/Mw tool (http://web.expasy.org/compute_pi/). Mega 7.0 was used to construct a phylogenetic tree based on the neighbor-joining method. DNAMAN were used to determine nucleotide substitutions and the conservation of functional sites.

### RNA isolation and expression analysis

Total RNA extraction and cDNA synthesis were performed as previously described (Liu et al., 2016). The expression levels of *PsCAT1*, *PsCAT2* and *PsCAT3* were measured during *Pst* infection of wheat by semi-quantitative RT-PCR. Elongation factor-1 (EF-1) from *Pst* was used as an internal reference (Liu et al., 2016). Semi-quantitative RT-PCR was conducted using 40 cycles of 95°C for 30 s, 55°C for 30 s and 72°C for 5 s. The primers used for semi-quantitative RT-PCR are listed in Supporting Information Table S1.

### Plasmid construction

To biochemically characterize PsCAT1, the coding region sequences (CDS) of *PsCAT1* without the signal peptide sequences was amplified and cloned into the *EcoR*I/*Xho*I restriction sites of vector pET15b-SUMO to obtain the recombinant plasmid pET15b-SUMO-*PsCAT1*.

To determine polymerization of PsCAT1, the CDS of *PsCAT1* without the signal peptide sequences was inserted into the *EcoR*I/*BamH*I sites of pGADT7 and pGBKT7 to generate the recombinant constructs pGADT7-*PsCAT1* and pGBKT7-*PsCAT1*, respectively. In addition, *PsCAT1* without the signal peptide sequences was also cloned into the *BamH*I restriction site in the binary vectors pSPYNE(R)173 and pSPYCE(M) (Waadt et al., 2008) to generate the recombinant plasmids pSPYCE(M)-*PsCAT1* and pSPYNE(R)173-*PsCAT1*, respectively.

To confirm the function of the identified signal peptide of PsCAT1, a yeast secretion system was established. The yeast signal trap vector pSUC2T7M13ORI (pSUC2), which carries a truncated invertase, SUC2, lacking both its initiation methionine and signal peptide, was used. DNA fragments encoding the predicted signal peptide of PsCAT1 was inserted into the *EcoR*I/*Xho*I restriction sites of vector pSUC2. The signal peptides of Avr1b (positive controls), and the sequence encoding the first 25 amino acids of Mg87 (negative control) were generated the recombinant plasmids pSUC2-*Avr1b*_*sp*_ and pSUC2-*Mg87*_*1–75*_ (Gu et al., 2011).

To identify the function of *PsCAT1*, the CDS of *PsCAT1* sequences was amplified and inserted into the *Not*I/*BamH*I restriction sites in plasmid pDR195 to obtain the complementation construct pDR195-*PsCAT1*.

For transient expression of *PsCAT1* in tobacco, the CDS of *PsCAT1* without a signal peptide and the *Bax* gene were PCR-amplified and inserted into the *Cla*I/*Not*I restriction sites in vector potato virus X (PVX) to obtain the recombinant plasmids PVX-*PsCAT1* and PVX-*Bax*, respectively.

Construction of the recombinant BSMV-HIGS vectors was performed as described by Holzberg et al. (2002). To specifically silence the PsCAT1 gene, the two γ RNA-based derivative plasmids BSMV-*PsCAT1*as1 and BSMV-*PsCAT1*as2 were constructed using a 142-bp fragment and a 133-bp fragment, which exhibited the highest polymorphism in the CAT gene family of *Pst* and the lowest nucleotide sequence similarity with other genes from *Pst* and wheat.

The primers used for all constructs are listed in Table S1.

### Expression and enzymatic characterization of PsCAT1

The *PsCAT1* gene was amplified by PCR using a *Pst*-infected wheat cDNA sample as a template. The constructed recombinant plasmid pET15b-sumo-*PsCAT1* was transformed into *E. coli* BL21(DE3) plysS and then protein expression was induced by 0.4 mM IPTG supplement overnight at 16°C. The collected cells were suspended in ice-cold phosphate-buffered saline (PBS) solution and lysed by sonication.

The supernatant containing the soluble proteins was analyzed by SDS-PAGE, and protein purification was performed using a HisTrap FF affinity column (GE Healthcare, Uppsala, Sweden), and identified by Western blotting analysis as described by Liu et al. (2016).

CAT activity was assayed using Catalase Assay Kit (Beyotime Biotechnology, China, Beijing) according to the manufacturer’s instructions. The optimum temperature and pH of the purified enzyme were determined after the enzyme solution samples were incubated at 20 to 70°C and pH 3 to 13 for 30 min, respectively. The effects of four metal ions (0.1 mM Mn^2+^, Zn^2+^, Cu^2+^, or Fe^2+^) on the CAT activity were measured. In addition, the dynamic curve was constructed to determine *K*m and *V*max values. All assays were repeated three times.

### Size-exclusion chromatography analysis of PsCAT1

The PsCAT1 protein sample was purified by nickel ion affinity chromatography, dialysis to remove salt, and cation exchange chromatography. The native molecular weight of PsCAT1 was then determined using size exclusion chromatography. The samples were loaded into a Superdex™200 column (GE Healthcare) equilibrated in 50 mM PBS, pH 7.4, at a flow rate of 0.5 ml min^− 1^ for preparative-scale fractionation. Protein fractions were collected based on UV absorbance at 280 nm and the elution times. The column was calibrated by chromatographic protein standards (thyroglobulin, 669 kDa; globulin, 158 kDa; ovalbumin, 44 kDa; carbonic anhydrase, 29 kDa; ribonuclease A 13.7 kDa).

### Yeast two-hybrid assays

Self-interactions of PsCAT1 were investigated by co-transformation of the recombinant plasmids pGBKT7-*PsCAT1* and pGADT7-*PsCAT1* into the yeast strain AH109. Transformed cells were cultured on SD (synthetic dropout)-Leu-Trp (SD-LW) and SD-Leu-Trp-His (SD-LWH) media at 30 °C for 3 days. A single colony was cultured and serial 1:10 dilutions were plated in either SD-Leu-Trp-His-Ade (SD-LWHA) or SD-LWHA containing X-α-Gal media. Cell growth was observed 3 days after plating.

Co-transformations with pGBKT7-Lam and pGADT7-T, pGADT7 and pGBKT7-*PsCAT1*, and pGBKT7 and pGADT7-*PsCAT1* were used as the negative controls, whereas Co-transformation with pGBKT7–53 and pGADT7-T was used as the positive control.

### BiFC assay in *N. benthamiana*

The recombinant plasmids pSPYNE(R)173-*PsCAT1* or pSPYCE(M)-*PsCAT1* were individually transformed into *A. tumefaciens* strain GV3101 and co-infiltrated into *N. benthamiana* leaves. After 48 h, self-interaction of PsCAT1 was determined by monitoring yellow fluorescent protein (YFP) signal by confocal microscopy, with an excitation laser at 488 nm. Yn:TaSGT1 and TaRAR1:Yc were used as the positive control (Wang et al., 2015). All of the assays were repeated independently at least three times with comparable results.

### Functional validation of the signal peptide

To confirm the function of the predicted signal peptide of PsCAT1, the yeast signal sequence trap system was used as described previously (Yin et al., 2018). The recombinant vector pSUC2-*PsCAT1*_*SP*_ was transformed into the invertase mutant yeast strain YTK12 (Oh et al., 2009). In this experiment, the signal peptides of Avr1b form *Phytophthora sojae* was used as positive controls, and the first 25 amino acids of a non-secreted protein from *M. oryzae* Mg87 was used as a negative control (Gu et al., 2011). CMD-W medium (0.67% yeast nitrogen base (YNB) without amino acids, 0.075% tryptophan dropout supplement, 2% sucrose, 0.1% glucose and 2% agar) and YPRAA medium (1% yeast extract, 2% peptone, 2% raffinose, 2 mg ml^− 1^ antimycin A and 2% agar) were prepared to determine the secretory function of the signal peptide. Moreover, invertase enzymatic activity was detected by the reduction of TTC to insoluble red colored TPF according to procedures and conditions described previously (Zheng et al., 2020).

### Heterologous expression of *PsCAT1* in *S. cerevisiae*

To identify the function of *PsCAT1*, the recombinant plasmids pDR195-*PsCAT1* and pDR195 were transformed into a *S. cerevisiae* strain YNL24C, respectively. The positive transformants carrying the pDR195-*PsCAT1* vector were confirmed by PCR analysis. Then, the function of *PsCAT1* was determined based on the growth of the positive transformants in SC media with different concentrations of H_2_O_2_. Growth was monitored in SC with different carbon sources as previously described by Longo et al. (1996). Yeast cells grown in SC without uracil were standardized to 1 × 10^7^ cells/ml. Five microliter volumes of a 10-fold dilution series were then spotted on the surface of SC agar plates. The plates were incubated and cell growth was observed for 48 h at 30 °C. The *S. cerevisiae* strain containing the empty pDR195 vector was used as the control.

### *A. tumefaciens* mediated transient expression of *PsCAT1*

The *A. tumefaciens*-mediated transient expression method was used to assay suppression of Bax- induced cell death by PsCAT1. The recombinant plasmids PVX-HA-*PsCAT1*, PVX-HA-*GFP* and PVX-*Bax* were transformed into *A. tumefaciens* strain GV3101, respectively. The confirmed positive *A. tumefaciens* cells carrying PVX-HA-*PsCAT1* and PVX-HA-*GFP* at a final OD600 of 0.4 and 10 mM MgCl_2_ buffer were infiltrated into *N. benthamiana* leaves. After 24 h, the same infiltration site was challenged with *A. tumefaciens* cell suspensions carrying the Bax gene. The infected leaves were harvested to extract total proteins for Western blotting analysis at 72 hpi. After 6 to 8 dpi, symptoms of the leaves were monitored and recorded.

In addition, the infected leaves were stained by trypan blue and DAB respectively. The leaves were then photographed after decolorized and transparentized.

### BSMV-mediated *PsCAT1* silencing in the compatible wheat-*Pst* interaction

For silencing of *PsCAT1*, construction of the recombinant vectors (*γ-PsCAT1*-as1 and *γ*-*PsCAT1*-as2) viral inoculation were performed as described previously by Holzberg et al. (2002). The wheat plants inoculated with BSMV-*TaPDS* (phytoene desaturase) were used as the positive control, whereas the BSMV-*γ*-inoculated plants were acted as the negative control. The wheat seedlings after inoculation were incubated in a plant growth chamber for 9–10 days at 25–27°C. Then, the fourth leaves were further infected with fresh CYR31 urediospores (for silencing of *PsCAT1*), and sampled at 0,12, 24, 48, 72 and 120 hpi for silencing efficiency calculations and histological observation (Wang et al., 2007). Fungal biomass was measured as previously described (Liu et al., 2016). The infected leaves were assessed for phenotype identification and photographed at 15 dpi. Biological replicates were carried out in triplicate.

### Histological observation of fungal growth and host response

To characterize the function of *PsCAT1* in the wheat-*Pst* infections, the fungal development and host response were observed by microscopy. The leaf segments were fixed and stained as described in Wang et al. (2007). H_2_O_2_ accumulation was detected by staining with DAB (Amresco, Solon, OH, USA). The *Pst* infection structures were stained with wheat germ agglutinin (WGA) conjugated to Alexa Fluor-488 (Invitrogen, Carlsbad, CA, USA) and observed under blue-light excitation (excitation wavelength 450–480 nm, emission wavelength 515 nm) with a BX51 Microscope (Olympus). Haustorial mother cells, haustoria, hyphal length and branches as well as colony size were analyzed statistically as previously described (Liu et al., 2016). Only the infection site where an appressorium had formed over a stoma was considered to be a successful penetration. A minimum of 50 infection sites from five randomly selected leaf segments were detected for each treatment.

## Supplementary Information

**Additional file 1: Fig. S1.** Prediction of the signal peptide of PsCATs. (a) PsCAT1 (b) PsCAT2 (c) PsCAT3 (d) PsCAT4 were predicted using SignalP 4.1 (http://www.cbs.dtu.dk/services/SignalP/). **Fig. S2.** Transcription profile analysis of *PsCATs* during the early stage of *Pst* infection. The transcript levels of *PsCAT1*, *PsCAT2, PsCAT3* at non-germinated urediniospores, 12, 24, 36, 48 hip, were analyzed by semi-quantitative RT-PCR (sqRT-PCR) with *PstEF1* as control. U, urediniospore. **Fig. S3.** Prediction of conserved domains of PsCAT1. (a) The protein domains and (b) The metal binding and active sites of catalytic reaction of *PsCAT1* were predicted by Pfam (http://pfam.sanger.ac.uk/) and Uniport (https://www.uniprot.org/), respectively. **Fig. S4.** Phylogenetic analysis of PsCAT1 and selected homologous proteins from other fungi. The unrooted phylogram was constructed based on the NJ method. The confidence level for the groupings was estimated using 1000 bootstrap replicates. The numbers adjacent to the branch points indicate the percentage of replicates supporting each branch. **Fig. S5.** Determination of the molecular weight of PsCAT1 by chromatography. (a) Gel filtration verifies the site where UV absorption peaks of protein sample. (b) Western blotting analysis of the sample at the absorption peak. Lane 1 is before chromatography, lane 2 is after chromatography. (c) Verification of the existence form of PsCAT1 after depolymerization. (d) Western blotting analysis of sample at the absorption peak. The arrow indicates the protein PsCAT1. M, maker. **Fig. S6.** Transient expression of *PsCAT1* in *N. benthamiana*. (a) Five injection sites on tobacco leaves. 1, PsCAT1; 2, PsCAT1 + Bax (infiltration 24 h later); 3, empty vector; 4, empty vector + Bax (infiltration 24 h later); 5, Bax. (b) Western blotting analysis of protein expression in *N. benthamiana* through GFP, HA and Bax antibodies. Ponceau S staining of the membrane indicates equal loading of proteins. **Fig. S7.** HIGS of *PsCAT1* led to restricted fungal development. (a) The average number of HB, HMC and H showed no significant difference in HIGS plants infected by CYR31 compared with the control at 48 hpi. (b) The infection unit area at 24 and 48 hpi per infection unit in HIGS plants infected by CYR31 was similar to that of the control. Values represent the means ± standard errors of three independent samples. Differences were assessed using Student’s *t*-test. HMC, haustorial mother cell; IH, infection hypha; HB, hyphal branch; H, haustoria. **Table S1.** Oligonucleotides and strains used in this study.

## Data Availability

All data generated or analyzed during this study are included in this published article and its supplementary information files.

## Abbreviations

ROS: Reactive oxygen species
CAT: Catalase
SOD: Superoxide dismutase
HIGS: Host-induced gene silencing
APX: Ascorbate peroxidase
GPX: Glutathione peroxidase
PRX: Peroxiredoxin
GSH: Glutathione
sqRT-PCR: Semi-quantitative reverse transcription-PCR
qRT-PCR: Quantitative reverse transcription-PCR
ORF: Open reading frame
BiFC: Bimolecular fluorescence complementation
EF-1: Elongation factor-1
CDS: Coding region sequences
PBS: Phosphate-buffered saline
YNB: Yeast nitrogen base
TTC: 2,3,5-Triphenyltetrazolium chloride
TPF: 1,3,5-Triphenylformazan
SC: Synthetic complete
BSMV: Barley stripe mosaic virus
WGA: Wheat germ agglutinin
HB: Hyphal branches
HMC: Haustorial mother cells
H: Haustoria
PDS: Phytoene desaturase
PVX: potato virus X
