# Supplementary material for: A secreted catalase contributes to Puccinia striiformis resistance to host-derived oxidative stress
Source: Stress Biol. 2021 Dec 29;1(1):22. doi: 10.1007/s44154-021-00021-2 (PMC10441885; doi:10.1007/s44154-021-00021-2)
Supplement: Supplementary file 1 — Additional file 1: Fig. S1. Prediction of the signal peptide of PsCATs. (a) PsCAT1 (b) PsCAT2 (c) PsCAT3 (d) PsCAT4 were predicted using SignalP 4.1 (http://www.cbs.dtu.dk/services/SignalP/). Fig. S2. Transcription profile analysis of PsCATs during the early stage of Pst infection. The transcript levels of PsCAT1, PsCAT2, PsCAT3 at non-germinated urediniospores, 12, 24, 36, 48 hip, were analyzed by semi-quantitative RT-PCR (sqRT-PCR) with PstEF1 as control. U, urediniospore. Fig. S3. Prediction of conserved domains of PsCAT1. (a) The protein domains and (b) The metal binding and active sites of catalytic reaction of PsCAT1 were predicted by Pfam (http://pfam.sanger.ac.uk/) and Uniport (https://www.uniprot.org/), respectively. Fig. S4. Phylogenetic analysis of PsCAT1 and selected homologous proteins from other fungi. The unrooted phylogram was constructed based on the NJ method. The confidence level for the groupings was estimated using 1000 bootstrap replicates. The numbers adjacent to the branch points indicate the percentage of replicates supporting each branch. Fig. S5. Determination of the molecular weight of PsCAT1 by chromatography. (a) Gel filtration verifies the site where UV absorption peaks of protein sample. (b) Western blotting analysis of the sample at the absorption peak. Lane 1 is before chromatography, lane 2 is after chromatography. (c) Verification of the existence form of PsCAT1 after depolymerization. (d) Western blotting analysis of sample at the absorption peak. The arrow indicates the protein PsCAT1. M, maker. Fig. S6. Transient expression of PsCAT1 in N. benthamiana. (a) Five injection sites on tobacco leaves. 1, PsCAT1; 2, PsCAT1 + Bax (infiltration 24 h later); 3, empty vector; 4, empty vector + Bax (infiltration 24 h later); 5, Bax. (b) Western blotting analysis of protein expression in N. benthamiana through GFP, HA and Bax antibodies. Ponceau S staining of the membrane indicates equal loading of proteins. Fig. S7. HIGS of PsCAT1 led to r [file 44154_2021_21_MOESM1_ESM.docx]

**Supporting Information**

Article title: A secretory catalase contributes to *Puccinia striiformis* resistance to host-derived oxidative stress

Author: Yuan Pu, Qian Wenhao, Jiang Lihua, Jia Conghui, Ma Xiaoxuan, Zhensheng Kang, Jie Liu

Fig. S1 Prediction of the signal peptide of PsCATs.

Fig. S2 Transcription profile analysis of *PsCATs* **during the early stage of *Pst* infection**.

Fig. S3 Prediction of conserved domains of PsCAT1.

Fig. S4 Phylogenetic analysis of PsCAT1 and selected homologous proteins from other fungi.

Fig. S5 Determination of the molecular weight of PsCAT1 by chromatography.

Fig. S6 Transient expression of *PsCAT1* in *N. benthamiana*.

Fig. S7 HIGS of *PsCAT1* led to restricted fungal development.

Table. S1. Oligonucleotides and strains used in this study.

Fig. S1 Prediction of the signal peptide of PsCATs. (a) PsCAT1 (b) PsCAT2 (c) PsCAT3 (d) PsCAT4 were predicted using SignalP 4.1 (http://www.cbs. dtu.dk/services/SignalP/).


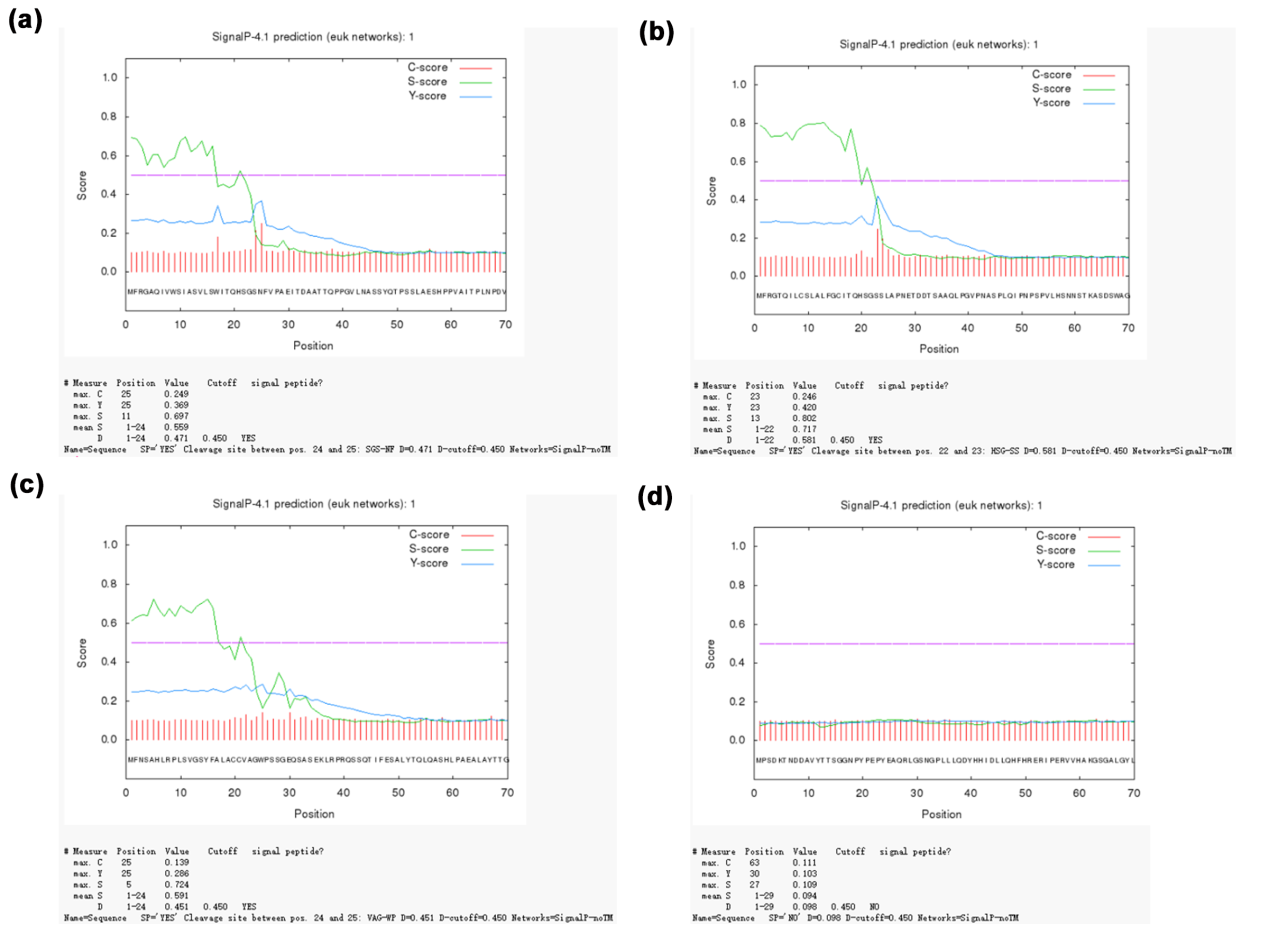


Fig. S2. Transcription profile analysis of *PsCATs* **during the early stage of *Pst* infection**. The transcript levels of *PsCAT1*, *PsCAT2, PsCAT3* at non-germinated urediniospores, 12, 24, 36, 48 hip, were analyzed by semi-quantitative RT-PCR (sqRT-PCR) with *PstEF1* as control. U, urediniospore.


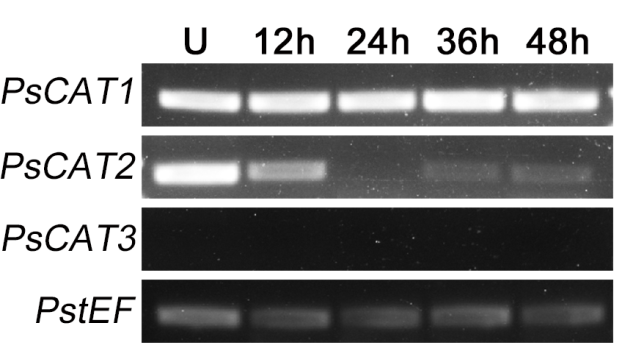


Fig. S3 Prediction of conserved domains of PsCAT1. (a) The protein domains and (b) The metal binding and active sites of catalytic reaction of *PsCAT1* were predicted by Pfam (<http://pfam.sanger.ac.uk/>) and Uniport (<https://www.uniprot.org/>), respectively.


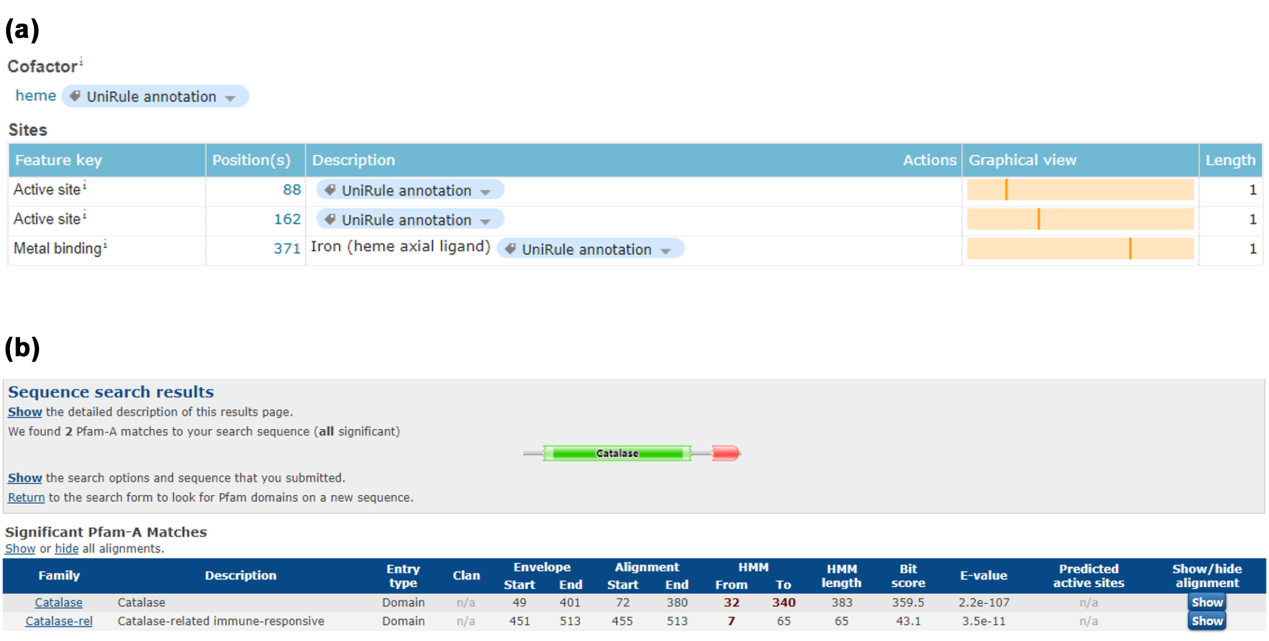


Fig. S4 Phylogenetic analysis of PsCAT1 and selected homologous proteins from other fungi. The unrooted phylogram was constructed based on the NJ method. The confidence level for the groupings was estimated using 1000 bootstrap replicates. The numbers adjacent to the branch points indicate the percentage of replicates supporting each branch.


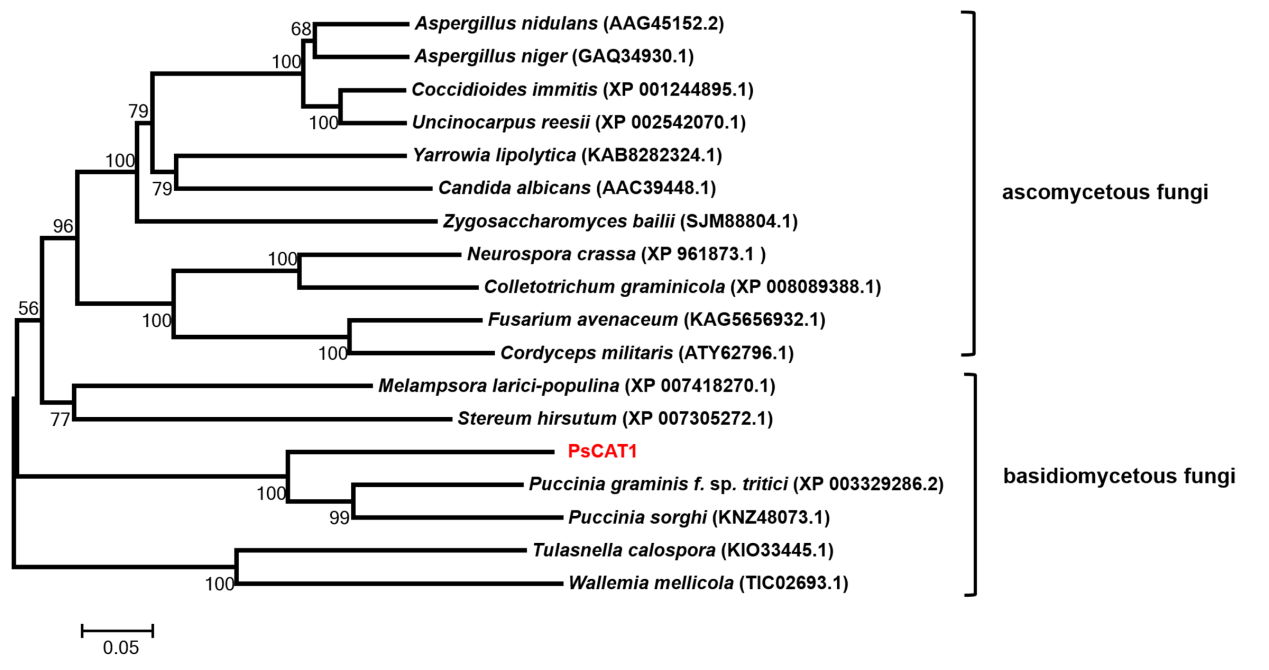


Fig. S5 Determination of the molecular weight of PsCAT1 by chromatography. (a). Gel filtration verifies the site where UV absorption peaks of protein sample. (b). Western blotting analysis of the sample at the absorption peak. lane 1 is before chromatography, lane 2 is after chromatography. (c). Verification of the existence form of PsCAT1 after depolymerization. (d). Western blotting analysis of sample at the absorption peak. lane 1 is the sample of 8.35 ml (a high polymer), and lane 2 is the sample of 14.69 ml (a monomer). The arrow indicates the protein PsCAT1. M, maker.


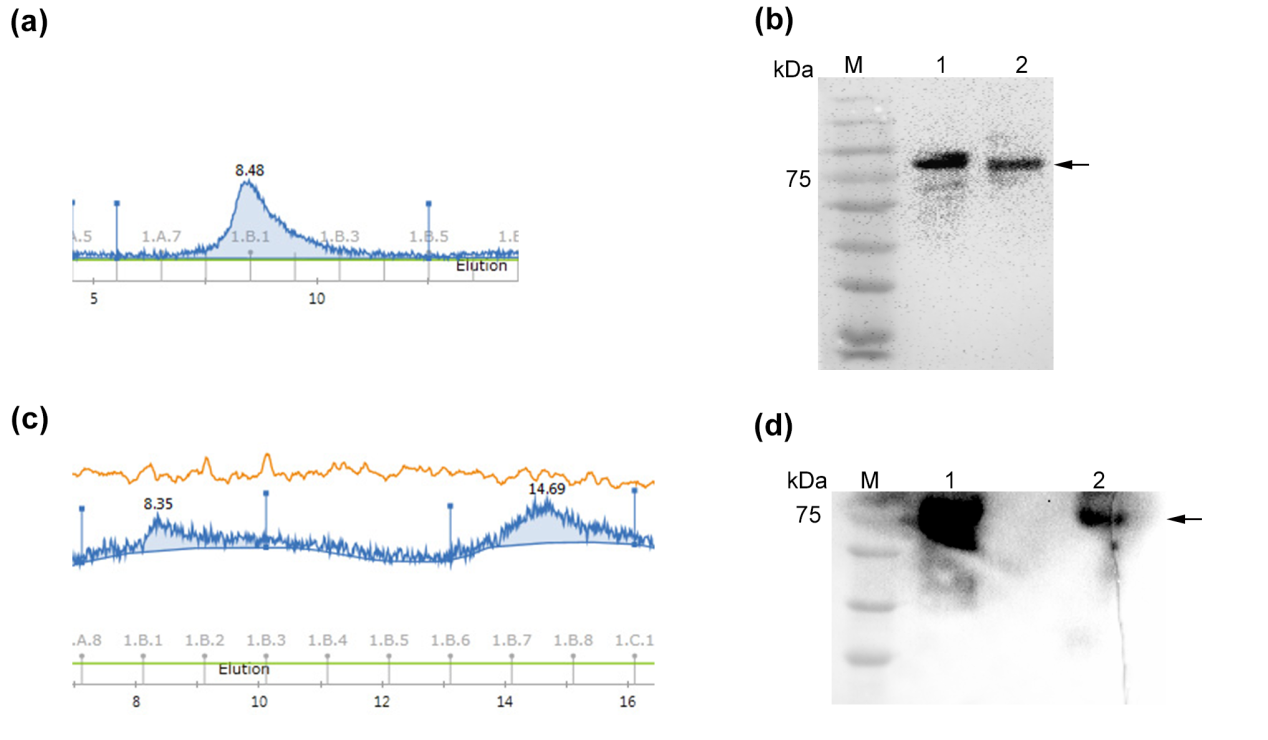


Fig. S6. Transient expression of *PsCAT1* in *N. benthamiana*. (a). Five injection sites on tobacco leaves. 1, PsCAT1; 2, PsCAT1 + Bax (infiltration 24 h later); 3, empty vector; 4, empty vector + Bax (infiltration 24 h later); 5, Bax. (b). Western blotting analysis of protein expression in *N. benthamiana* through GFP, HA and Bax antibodies. **Ponceau S staining of the membrane indicates equal loading of proteins.**


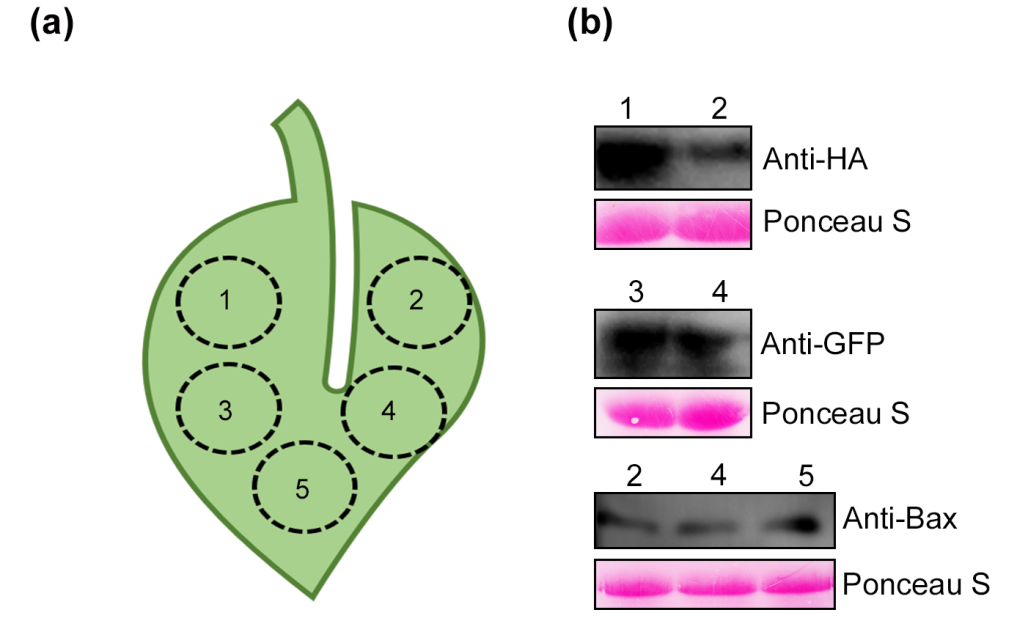


Fig. S7 HIGS of *PsCAT1* led to restricted fungal development. (a) The average number of HB, HMC and H showed no significant difference in HIGS plants infected by CYR31 compared with the control at 48 hpi. (b) The infection unit area at 24 and 48 hpi per infection unit in HIGS plants infected by CYR31 was similar to that of the control. Values represent the means ± standard errors of three independent samples. Differences were assessed using Student’s *t*-tests. HMC, haustorial mother cell; IH, infection hypha; HB, hyphal branch; H, haustoria.


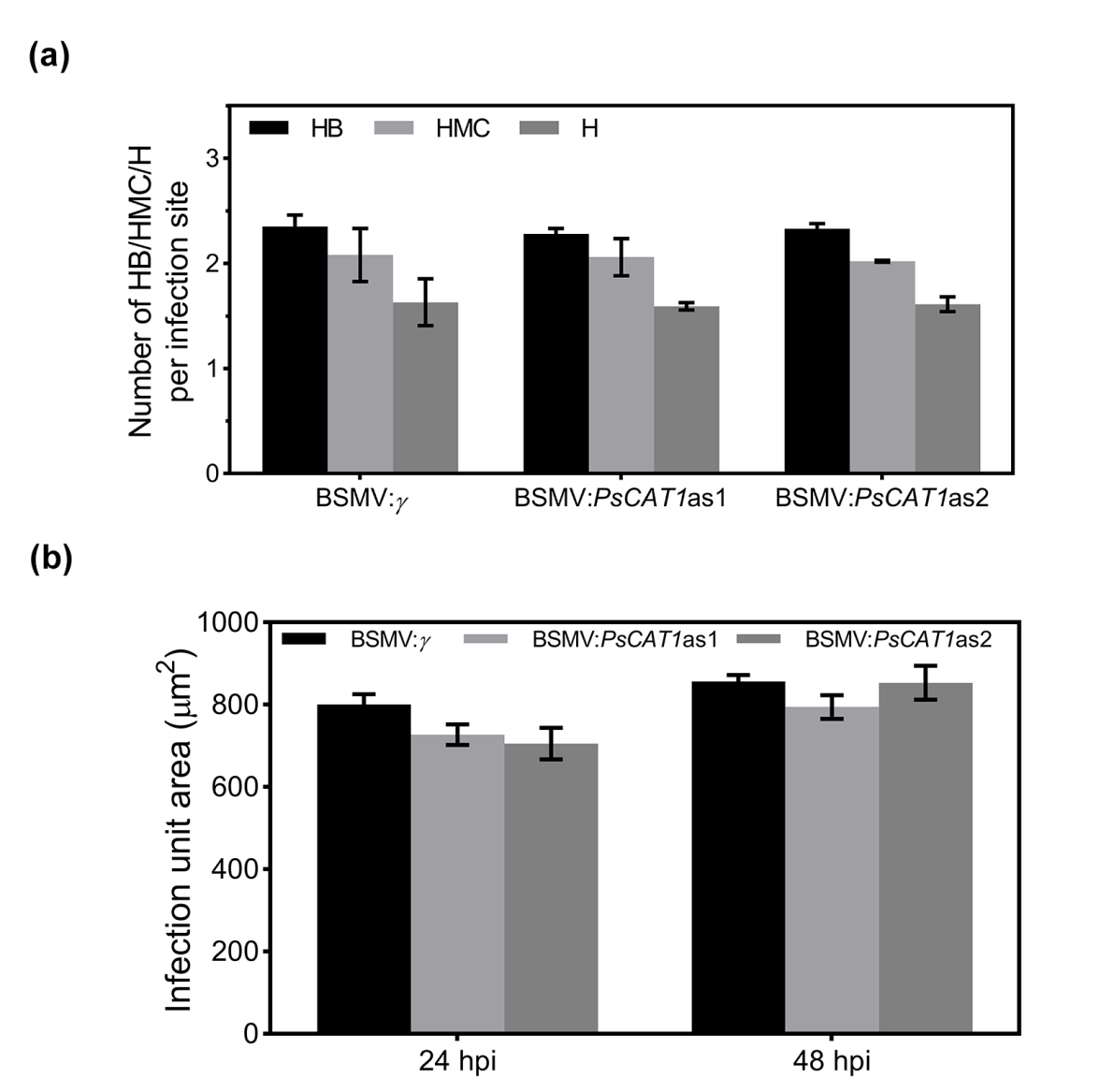


Table S1. Oligonucleotides and strains in this study

| Name | Sequence (5′—3′) | Application |
| --- | --- | --- |
| PsCAT1-F | ATGTTTCGGGGCGCTCAAATCGTCT | Amplification of cDNA of *PsCAT1* |
| PsCAT2-R | TCATCCGGTCATCGTCTTCAACTTC |  |
| PsCAT1-PET15b-F | cagattggcggcgaattcATGAACTTTGTGCCTGCT | Prokaryotic expression of *PsCAT1* |
| PsCAT1-PET15b-R | agccggttcctcgagTCCGGTCATCGTCTTCAA |  |
| PsCAT1-SP-PSUC2-F | cggaattttaattaagaattcATGTTTCGGGGCGCTCAA | Functional identification of signal peptides of PsCAT1 |
| PsCAT1-SP-PSUC2-R | cactatagggagaacctcgagACTTCCACTGTGTTGAGT |  |
| Mg87-SP-PSUC2-F | cggaattttaattaagaattcATGCCTGTTCCCTTCGAG | Functional identification of the predicted signal peptide of Mg87 |
| Mg87-SP-PSUC2-R | cactatagggagaacctcgagGATACCGGCACCCGAAATG |  |
| Avr1b-SP-PSUC2-F | cggaattttaattaagaattc ATGCGTCTATCTTTTGTGC | Functional identification of the predicted signal peptide of Avr1b |
| Avr1b-SP- PSUC2-R | cactatagggagaacctcgagAGTCATTGCGTTGCAGGTC |  |
| PsCAT1-YN-F | cccaggcctactagtggatcc ATGAACTTTGTGCCTGCT | Construction of pSPYNE(R)173-*PsCAT1* |
| PsCAT1-YN-R | accctcgaggtcgacggatccTCATCCGGTCATCGTCTTCAA |  |
| PsCAT1-YC-F | tggcgcgccactagtggatcc ATGAACTTTGTGCCTGCT | Construction of  pSPYCE(M)-*PsCAT1* |
| PsCAT1-YC-R | gacagtactatcgatggatccTCCGGTCATCGTCTTCAA |  |
| PsCAT1-AD-F | gccatggaggccagtgaattc ATGAACTTTGTGCCTGCT | Construction of pGADT7-*PsCAT1* |
| PsCAT1-AD-R | cagctccgagctcgatggatccTCATCCGGTCATCGTCTTCAA |  |
| PsCAT1-BD-F | atggccatggaggccgaatcc ATGAACTTTGTGCCTGCT | Construction of pGBKT7-*PsCAT1* |
| PsCAT1-BD-R | ccgctgcaggtcgacggatccTCATCCGGTCATCGTCTTCAA |  |
| PsCAT1-PVX107HA-F | agaggtcagcaccagctagcatcgatATGAACTTTGTGCCTGCT | Construction of pVX107HA- *PsCAT1* |
| PsCAT1-PVX107HA-R | gaacatcgtatgggtacgcggccgcTCCGGTCATCGTCTTCAA |  |
| PsCAT1-PDR- F | cagcctcgagggatccATGTTTCGGGGCGCTC | Construction of pDR195- *PsCAT1* |
| PsCAT1-PDR- R | gtccaaagctggatccTCAGTGGTGGTGGTGGTGGTG |  |
| PsCAT1-QRT-F | CCAGTAATCCTGAAAGTATGA | Quantitative RT-PCR of *PsCAT1* transcripts |
| PsCAT1-QRT-R | CCAGTAATCCTGAAAGTATGA |  |
| PsEF-F | TTCGCCGTCCGTGATATGAACAA | Calculation of fungal biomass |
| PsEF-R | ATGCGTATCATGGTGGTGGAGTGA |  |
| PsCAT1-HIGS-F1 | atattaattaa CGCTCAAATCGTCTG | Construction of pγ::*PsCAT1-as1* |
| PsCAT1-HIGS-R1 | tatgcggccgc TGGAGTTTGATACGA |  |
| PsCAT1-HIGS-F2 | ccttaattaa TGGCGGATTGAGCAG | Construction of pγ::*PsCAT1-as2* |
| PsCAT1-HIGS-R2 | ttgcggccgc ATTTGGAATCAATCA |  |
|  | | |
| Strains | Genotype/comment | Application |
| JM109 | *E. coli*; recA1, supE44, endA1, hsdR17, gyrA96, relA1, thi, Δ(lac-proAB) | Constructions of the plasmids |
| BL21(DE3)plysS | *E. coli*; F-, ompT hsdS B (rBB-mB-), gal, dcm, (DE3, pLysS, Cm^r^) | prokaryotic expression of *PsCAT1* |
| GV3101 | *A. tumefaciens*; Rif^r^, Gen^r^ | Transient expression of *PsCAT1* |
| YTK12 | A invertase secretion-deficient yeast strain | Signal peptide validation of PsCAT1 |

*Uppercase letters indicate bases that match the initial template.

Lower case letters indicate 5**′** extensions that do not match the initial template. Restriction sites introduced into the amplicons are underlined.
